# Negative Regulators of an RNAi-Heterochromatin Positive Feedback Loop Safeguard Somatic Genome Integrity in *Tetrahymena*

**DOI:** 10.1016/j.celrep.2017.02.024

**Published:** 2017-03-07

**Authors:** Jan H. Suhren, Tomoko Noto, Kensuke Kataoka, Shan Gao, Yifan Liu, Kazufumi Mochizuki

**Affiliations:** 1 Institute of Molecular Biotechnology of the Austrian Academy of Sciences, 1030 Vienna, Austria; 2 Institute of Human Genetics, CNRS-University of Montpellier UMR9002, 34396 Montpellier, France; 3 Pathology Department, University of Michigan, Ann Arbor, MI 48109, USA

**Keywords:** Heterochromatin, RNAi, small RNAs, Tetrahymena, DNA elimination, genome rearrangement

## Abstract

RNAi-mediated positive feedback loops are pivotal for the maintenance of heterochromatin, but how they are downregulated at heterochromatin-euchromatin borders is not well understood. In the ciliated protozoan *Tetrahymena*, heterochromatin is formed exclusively on the sequences that are removed from the somatic genome by programmed DNA elimination, and an RNAi-mediated feedback loop is important for assembling heterochromatin on the eliminated sequences. In this study, we show that the heterochromatin protein 1 (HP1)-like protein Coi6p, its interaction partners Coi7p and Lia5p, and the histone demethylase Jmj1p are crucial for confining the production of small RNAs and the formation of heterochromatin to the eliminated sequences. The loss of Coi6p, Coi7p, or Jmj1p causes ectopic DNA elimination. The results provide direct evidence for the existence of a dedicated mechanism that counteracts a positive feedback loop between RNAi and heterochromatin at heterochromatin-euchromatin borders to maintain the integrity of the somatic genome.

## Highlights

• The HP1-like protein Coi6p confines small RNA and heterochromatin formation
• Two Coi6p-binding proteins and the histone demethylase Jmj1p likely act with Coi6p
• Coi6p and Jmj1p are important for preventing ectopic DNA elimination
• Suppression of RNAi-heterochromatin feedback loop maintains somatic genome integrity

## Graphical Abstract

**Figure:**
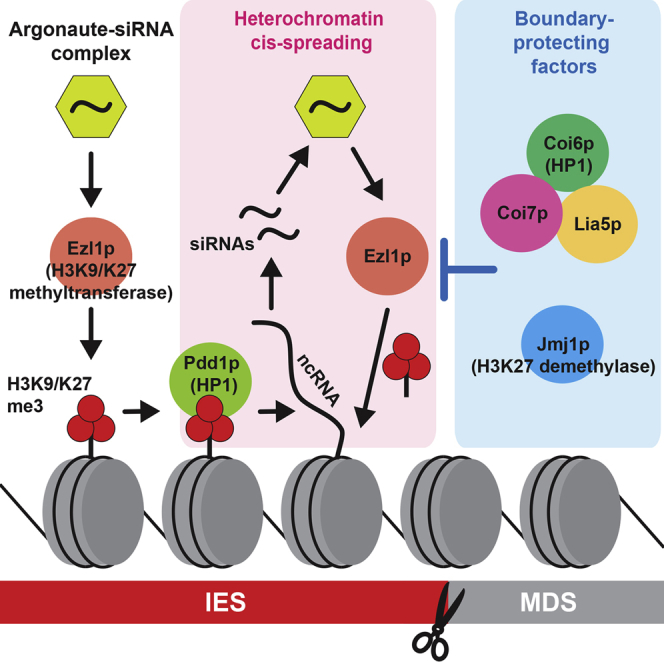

## Teaser

Regulation of a small RNA-mediated positive feedback loop is pivotal for confining heterochromatin to proper locations. Suhren et al. show the existence of a dedicated mechanism that counteracts a small RNA-heterochromatin positive feedback loop at heterochromatin-euchromatin borders to maintain the integrity of the somatic genome in *Tetrahymena*.

## Introduction

Heterochromatin is a compacted region of eukaryotic chromosomes that is used for various genome regulations, such as chromosome segregation and gene silencing, and RNAi mechanisms play a key role in the assembly of heterochromatin in several eukaryotes (Grewal, 2010, Martienssen and Moazed, 2015). In the fission yeast *Schizosaccharomyces pombe*, small interfering RNAs (siRNAs) are produced from repeats at major heterochromatic loci and target the Argonaute protein Ago1 to nascent transcripts from these repeats. This interaction recruits the methyltransferase Clr4, which promotes the accumulation of methylated histone H3 lysine 9 (H3K9me) and its binders, the heterochromatin protein 1 (HP1) homologs Swi6 and Chp2, thereby establishing heterochromatin (Grewal, 2010). Ago1 and Swi6 also recruit the RNA-dependent RNA-polymerase and the Dicer protein (Hayashi et al., 2012, Rougemaille et al., 2012, Sugiyama et al., 2005), resulting in the further production of siRNAs, which constitutes a self-reinforcing feedback loop for heterochromatin maintenance. Similar feedback loops are operational in piwi-associated RNA (piRNA)-mediated transcriptional silencing in the fruit fly *Drosophila melanogaster*, in which the HP1 homolog Rhino promotes the production of piRNAs at the targeted loci (Mohn et al., 2014, Zhang et al., 2014), and in RNA-directed DNA methylation in the flowering plant *Arabidopsis thaliana*, in which DNA methylation and H3K9me cooperate to reinforce the RNAi signal (Pikaard and Mittelsten Scheid, 2014).

Although the RNAi-mediated feedback loop is pivotal for the maintenance of heterochromatin, it must be stalled at heterochromatin borders to avoid abnormal genome regulation (Talbert and Henikoff, 2006). At the centromeres and the silent mating type locus of fission yeast, B-box sequences of tRNA genes prevent the spreading of heterochromatin by recruiting the transcription factor TFIIIC, which tethers the boundaries to the nuclear periphery (Noma et al., 2006, Scott et al., 2007). Similarly, insulator elements establish structural barriers for heterochromatin boundaries in mammals (Cuddapah et al., 2009, Narendra et al., 2015). In contrast, one side of the fission yeast centromere 1 lacks a B-box but contains inverted repeat elements, which show preferential enrichment of a JmjC protein Epe1 known to prevent spreading of heterochromatin into neighboring sequences (Noma et al., 2006, Zofall and Grewal, 2006). This region also expresses a noncoding RNA that evicts Swi6 to prevent heterochromatin spreading (Keller et al., 2013). Therefore, localized activities preventing heterochromatin formation also play important roles in the maintenance of heterochromatin boundaries. RNAi-mediated heterochromatin spreading may also be interrupted by inhibiting the production or action of small RNAs, although such a mode of regulation has not been reported.

**Figure 1:**
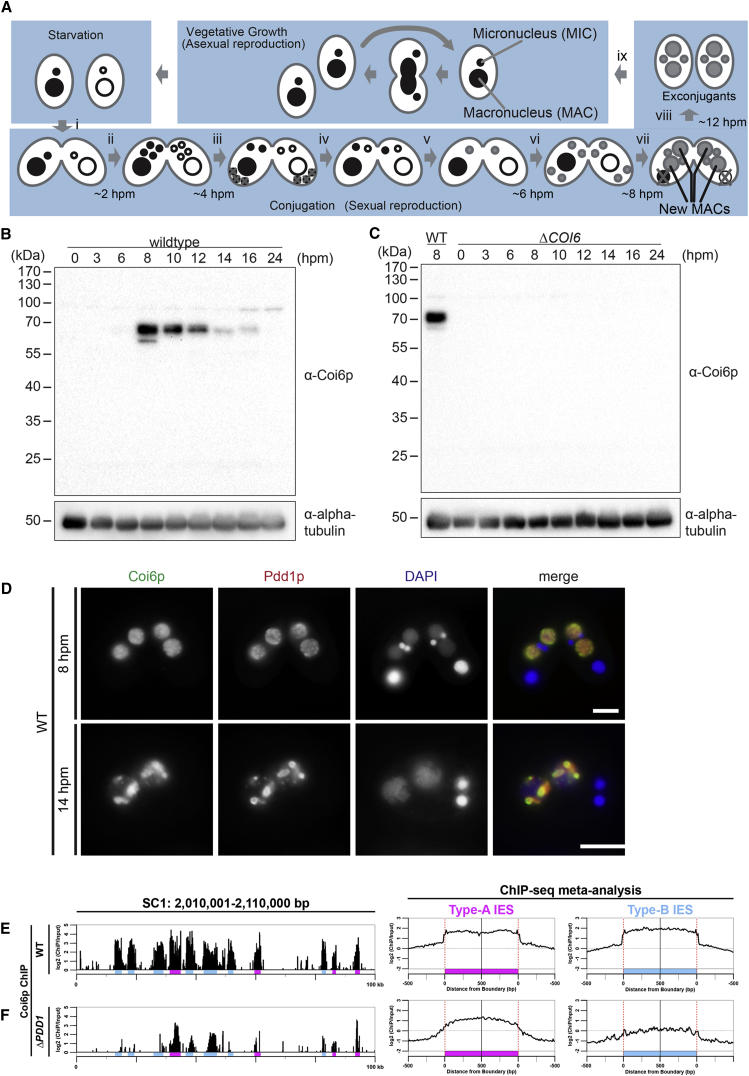
Coi6p Associates with IESs (A) Life cycle of *Tetrahymena*. Each cell contains a macronucleus (MAC) and a micronucleus (MIC), both of which divide and segregate to daughter cells during vegetative growth. Mixing starved cells of different mating types induces conjugation (i). The MICs undergo meiosis (ii), and one of the meiotic products divides mitotically to form two pronuclei (iii). One of the pronuclei crosses the conjugation bridge (iv) and fuses with the stationary pronucleus to produce the zygotic nucleus (v), which then divides twice (vi) to form two new MACs and two MICs (vii). The parental MAC is degraded, and the pair is dissolved (viii). The exconjugants resume vegetative growth upon nutrient supply (ix). The approximate time when each event occurs is indicated. hpm, hours post-mixing. (B and C) The proteins from WT (B) and *ΔCOI6* cells (C) at the indicated time points of conjugation were analyzed by western blot using the anti-Coi6p and an anti-alpha-tubulin antibody. (D) The cytological localizations of Coi6p and Pdd1p in WT cells at 8 and 14 hpm were analyzed by indirect immunofluorescent staining using the rabbit anti-Coi6p and a guinea pig anti-Pdd1p antibody, respectively. DNA was stained with DAPI. Scale bars, 10 μm. (E and F) Chromosomal localizations of Coi6p in WT (E) and *ΔPDD1* (F) cells at 12 hpm were analyzed by ChIP-seq using the anti-Coi6p antibody. Sequence reads were mapped to a 100-kb genomic region with 100-bp bins (left) or to compiled 500-bp sequences inside and outside of the boundaries of type-A and type-B IESs with 10-bp bins (right), and the mapped and normalized read numbers from ChIP-seq were divided by the corresponding numbers from input. Type-A and type-B IESs were marked in magenta and blue, respectively. See also Figure S1.

RNAi mechanisms underlie the programmed DNA elimination of ciliated protists (Fang et al., 2012, Mochizuki et al., 2002, Sandoval et al., 2014), and an RNAi-heterochromatin feedback loop acts in this process in *Tetrahymena thermophila* (Noto et al., 2015). *Tetrahymena* has two distinct nuclei in a single cell: the germline micronucleus (MIC) and the somatic macronucleus (MAC). During the conjugation (sexual reproduction) of *Tetrahymena*, new MICs and MACs are generated from a zygotic product of the MICs, while the parental MAC is degraded (Figure 1A). Then, in the new MAC, ∼10,000 internal eliminated sequences (IESs), which comprise one-third of the MIC genome and many of which are transposon related, are reproducibly removed (Hamilton et al., 2016). At early conjugation stages (∼2–3 hr post-mixing [hpm]), ∼60% of IESs, called type-A IESs, and their genomic surrounding are bi-directionally transcribed in the MIC, and the transcripts are processed to ∼26- to 32-nt siRNAs called Early-scnRNAs (Malone et al., 2005, Mochizuki and Gorovsky, 2005). Early-scnRNAs are loaded into the Argonaute protein Twi1p and move into the parental MAC, where those complementary to the MAC genome (i.e., non-IES sequences) are degraded (Aronica et al., 2008, Schoeberl et al., 2012). The remaining IES-specific Early-scnRNAs are then shuttled into the developing new MACs (∼7–8 hpm) and are believed to base pair with nascent transcripts from type-A and type-B IESs, the latter of which constitute ∼40% of all IESs and share repetitive sequences with type-A IESs (Noto et al., 2015). These interactions recruit the methyltransferase Ezl1p, which mediates the accumulation of H3K9me, H3K27me, and the HP1-like protein Pdd1p to IESs (Liu et al., 2007). This heterochromatin nucleation induces the production of Late-scnRNAs, additional ∼26- to 32-nt siRNAs that further promote heterochromatin assembly, and thus form a positive feedback loop (Noto et al., 2015). A heterochromatin-binding endonuclease eventually excises IESs, and their flanks are ligated at ∼12–16 hpm (Cheng et al., 2010, Lin et al., 2012, Vogt and Mochizuki, 2013).

Because heterochromatin is specifically formed on IESs (Kataoka and Mochizuki, 2015) and Late-scnRNAs are exclusively derived from IESs (Noto et al., 2015), some mechanism must inhibit the RNAi-heterochromatin feedback loop at the boundaries of IESs in *Tetrahymena*. Here, we present genetic evidence for the presence of a mechanism forming precise heterochromatin boundaries at IESs and its importance in accurate DNA elimination in *Tetrahymena*.

## Results

### Coi6p Is an HP1-like Protein Associated with Heterochromatin in the New MAC

*COI6* is a conjugation-induced (*COI*) gene that encodes the HP1-like protein Coi6p (Figures S1A and S1B) and is important for DNA elimination (Woehrer et al., 2015). To localize Coi6p, we raised an antibody against recombinant Coi6p. Western blot analysis showed that this antibody recognized a protein migrating at ∼65 kDa that was expressed exclusively at late conjugation stages (8–16 hpm) in wild-type (WT) cells (Figure 1B). This agrees well with the predicted molecular weight of Coi6p (60 kDa) and the late conjugation-specific expression of *COI6* mRNA (Figure S1C). The protein was not detected in *COI6* knockout (KO; *ΔCOI6*) cells (Figure 1C), in which all copies of the *COI6* gene in both MIC and MAC were disrupted (Woehrer et al., 2015) (Figure S1D). Altogether, we conclude that this antibody specifically recognizes Coi6p.

Immunofluorescent staining using this antibody showed that in the new MACs of WT cells, Coi6p was localized homogeneously at 8 hpm and in foci at 14 hpm (Figure 1D). These foci were heterochromatin bodies in which heterochromatinized IESs accumulated because they also contained Pdd1p (Figure 1D), the other HP1-like protein that is known to localize in heterochromatin bodies (Kataoka and Mochizuki, 2015, Taverna et al., 2002). Therefore, Coi6p is a component of heterochromatin in the new MAC.

### Coi6p Accumulates on IESs

**Figure 2:**
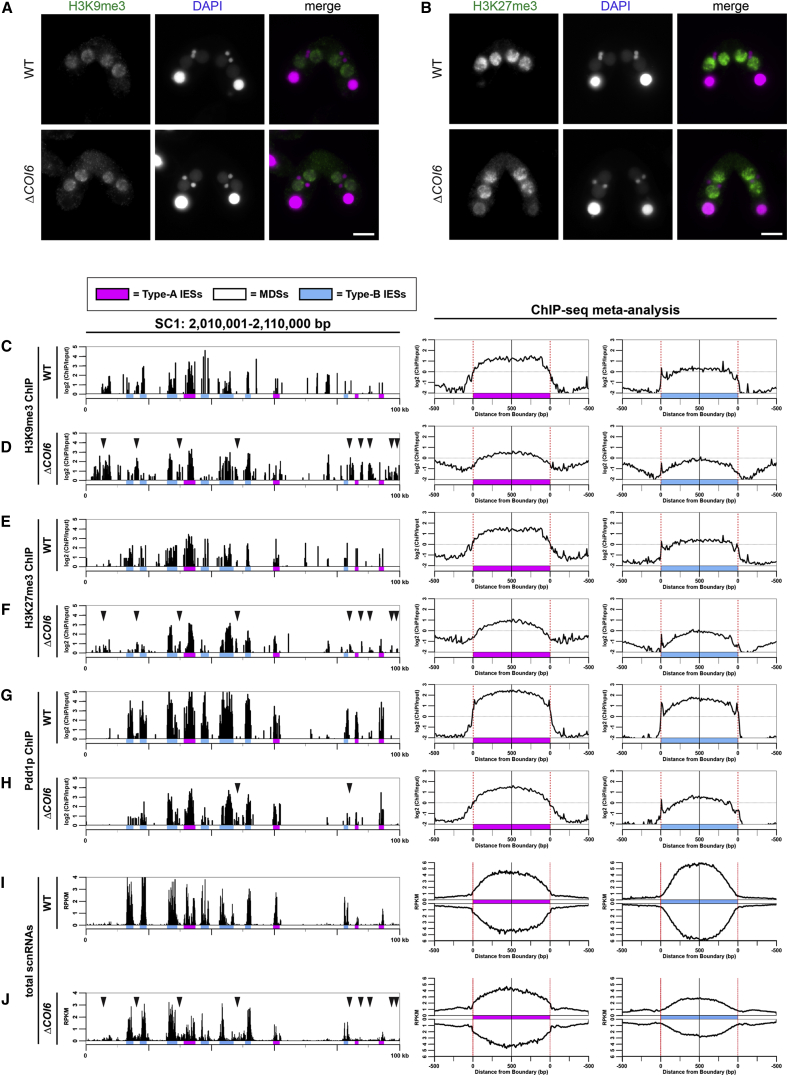
Coi6p Confines Heterochromatin within IESs (A and B) The localization of H3K9me3 (A) and H3K27me3 (B) in wild-type (WT, top) and *COI6* KO (*ΔCOI6,* bottom) cells at 8 hpm was analyzed by indirect immunofluorescent staining using an anti-H3K9me3 and an anti-H3K27me3 antibody, respectively. DNA was counterstained with DAPI. Scale bars, 10 μm. (C–H) The chromosomal localizations of H3K9me3 (C and D), H3K27me3 (E and F), and Pdd1p (G and H) in WT (C, E, and H) and *ΔCOI6* (D, F, and H) cells at 12 hpm were analyzed by ChIP-seq and analyzed as in Figure 1E. Arrowheads indicate regions in which the ectopic accumulation of the corresponding molecules was detected in *ΔCOI6* cells. (I and J) Small RNAs from WT (I) and *ΔCOI6* (J) cells at 12 hpm were sequenced, and 26- to 32-nt RNAs (scnRNAs) were mapped to a 100-kb genomic region and to the compiled 500-bp sequences inside and outside of the boundaries of type-A and type-B IESs with 10-bp bins. The numbers of sense and anti-sense strand mapped scnRNAs are shown on the top and bottom of each graph, respectively.

We next analyzed the chromosomal localization of Coi6p. At 12 hpm, a stage at which heterochromatin formation is largely completed, but most IESs remain in the new MAC chromosomes, the new MACs were enriched by fluorescence-activated sorting from WT cells and used for ChIP-seq (chromatin immunoprecipitation followed by DNA sequencing) with the anti-Coi6p antibody. The MIC genome (and the new MAC genome prior to DNA elimination) mainly consists of three types of sequences: type-A and type-B IESs and MAC-destined sequences (MDSs) that lie between IESs (Noto et al., 2015). We found that Coi6p accumulated on most of the type-A and type-B IESs in a representative 100-kb MIC locus (Figure 1E, left, magenta and blue, respectively). A meta-analysis for the compiled 500 bp up- and downstream of the boundaries of type-A and type-B IESs across the genome also showed that Coi6p was enriched on both type-A and type-B IESs (Figure 1E, right). ChIP-seq analysis using an anti-Pdd1p antibody (see Figure 2G) indicated that Pdd1p, the known heterochromatin component, localized similarly to Coi6p. These results indicate that Coi6p associates with IESs, regardless of their types.

We then asked whether the accumulation of Coi6p on IESs depends on Pdd1p. In the new MAC of *ΔPDD1* cells, Coi6p was enriched on type-A IESs, whereas its relative accumulation on type-B IESs was markedly reduced (Figure 1F). We previously demonstrated that the DNA elimination of the majority of type-A IESs only requires Early-scnRNAs, which are produced in a Pdd1p-independent manner, whereas the DNA elimination of many type-B IESs requires both Early- and Late-scnRNAs, of which the latter require Pdd1p for their production (Noto et al., 2015). Therefore, the most probable explanation for the above observations is that heterochromatin (or incomplete heterochromatin) on type-A IESs induced by Early-scnRNAs in the absence of Pdd1p is sufficient to recruit Coi6p, whereas such heterochromatin is insufficiently formed on type-B IESs because of a lack of Late-scnRNAs in *ΔPDD1* cells. These results indicate that Coi6p is an RNAi-dependent heterochromatin component on IESs.

### Coi6p Is Important for Confining Heterochromatin to IESs

We next asked whether Coi6p plays a role in the formation of heterochromatin. Immunofluorescent stainings showed that H3K9me3, H3K27me3, and Pdd1p similarly accumulated in the new MACs of WT and *ΔCOI6* cells at 8 hpm (Figures 2A, 2B, and S2G). However, ChIP-seq analysis at 12 hpm revealed that although H3K9me3 and H3K27me3 accumulated on both type-A and type-B IESs in *ΔCOI6* cells, they were less enriched compared with WT cells (Figures 2C–2F) and were also detected in some MDS regions (arrowheads in Figures 2D and 2F). This redistribution was also visible in the meta-analysis: whereas the localization of H3K9/K27me3 in the WT cells was sharply confined to IESs, it was distributed in a broader fashion in *ΔCOI6* cells (Figures 2C–2F, right).

Consistent with the fact that Pdd1p binds to H3K9me3 and H3K27me3 (Liu et al., 2007, Taverna et al., 2002), ChIP-seq analysis revealed that Pdd1p was also redistributed in *ΔCOI6* cells (Figures 2G and 2H). However, the redistribution of Pdd1p was less prominent than that of H3K9me3 and H3K27me3 in the absence of Coi6p (cf. Figure 2H with 2D and 2F), indicating that the localization of Pdd1p is not merely determined by the presence of H3K9/K27me3, but is also regulated by additional factors, which will be discussed below. Nonetheless, the results above indicate that Coi6p is not required for heterochromatin formation per se but is required for the precise accumulation of heterochromatin components on IESs.

### Coi6p Is Dispensable for the Biogenesis and Turnover of Early-scnRNAs

**Figure 3:**
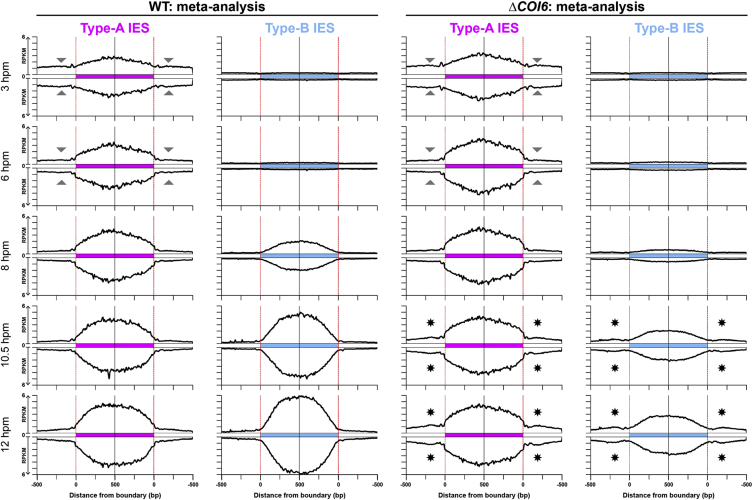
Coi6p Confines Late-scnRNA Production within IESs Small RNAs from WT (left) and *ΔCOI6* (right) cells at the indicated time points of conjugation were sequenced, and 26- to 32-nt RNAs (scnRNAs) were mapped as in Figure 2I. Arrowheads indicate MDS regions to which the amount of Early-scnRNAs mapped decreased between 3 and 6 hpm because of the selective degradation of Early-scnRNAs (“scnRNA selection”). Asterisks indicate MDS regions to which the amount of small RNAs mapped increased after 8 hpm because of the ectopic production of Late-scnRNAs.

Early-scnRNAs are 26- to 32-nt RNAs that are produced from the MIC at early conjugation stages (∼2–4.5 hpm) and are continuously present until later stages in WT cells (Noto et al., 2015). Because heterochromatin formation depends on Early-scnRNAs (Liu et al., 2004), we next analyzed Early-scnRNAs in the absence of Coi6p. Small RNAs at different stages of conjugation were sequenced, and 26- to 32-nt RNAs were mapped to compiled IES loci, as with the meta-analysis for ChIP-seq above. As we previously reported (Noto et al., 2015), in WT cells (Figure 3, WT), Early-scnRNAs at 3 hpm were mostly derived from type-A IESs and their surrounding MDS regions. At 6 hpm, they mapped more exclusively to type-A IESs (note that fewer RNAs were mapped to the regions marked by arrowheads at 6 hpm than at 3 hpm in Figure 3) because of “scnRNA selection,” in which Early-scnRNAs complementary to the parental MAC genome were selectively degraded (Aronica et al., 2008, Schoeberl et al., 2012). In *ΔCOI6* cells, Early-scnRNA accumulation at 3 hpm and the reduction of those mapping to MDS regions by 6 hpm occurred normally (Figure 3, *ΔCOI6*). We therefore conclude that heterochromatin redistribution in the absence of Coi6p does not occur at the level of Early-scnRNAs.

### Coi6p Is Important for Confining Late-scnRNA Production to IESs

In addition to Early-scnRNAs, another class of 26- to 32-nt siRNAs called Late-scnRNAs is produced from the new MACs. Because heterochromatin formation and Late-scnRNA production are interdependent (Noto et al., 2015), we next analyzed Late-scnRNAs in *ΔCOI6* cells. We previously showed that in WT cells, whereas Early-scnRNAs are produced in the parental MIC exclusively from type-A IESs and their surrounding MDS regions at early conjugation (∼3–4.5 hpm), Late-scnRNAs are expressed from both type-A and type-B IESs in the new MACs at late conjugation stages (∼7 hpm or later) (Noto et al., 2015). Therefore, 26- to 32-nt RNAs that map to type-B IESs can be identified as Late-scnRNAs.

As previously shown in WT cells, Late-scnRNAs that map to type-B IESs accumulated after 8 hpm (Figure 3, WT, type-B IES, 8–12 hpm). They were derived almost exclusively from IESs. In contrast, in *ΔCOI6* cells, Late-scnRNAs from type-B IESs were reduced, but those mapping to their surrounding MDS regions were increased (Figure 3, *ΔCOI6*, type-B IES, 8–12 hpm). MDS-mapped 26- to 32-nt RNAs were also elevated at type-A IES loci in *ΔCOI6* cells (Figure 3, *ΔCOI6*, type-A IES, 8–12 hpm). Because MDS-mapped Early-scnRNAs around type-A IES mostly disappeared by 6 hpm because of scnRNA selection, the small RNAs from these MDS regions at the later stages in *ΔCOI6* cells were also Late-scnRNAs. Altogether, we conclude that Coi6p prevents the production of Late-scnRNAs from outside of both type-A and type-B IESs.

An analysis of the small RNA-seq profiles of individual loci in *ΔCOI6* cells at 12 hpm (Figure 2J) revealed that although a large number of IES loci produced Late-scnRNA from MDSs (arrowheads in Figure 2J), other IES loci were unaffected. Most of the MDS loci that ectopically produced Late-scnRNAs in *ΔCOI6* cells also ectopically accumulated H3K9me3 and H3K27me3 (cf. Figures 2D, 2F, and 2J), suggesting that Coi6p is important for stalling the *cis*-spreading of heterochromatin and Late-scnRNA production by downregulating the RNAi-heterochromatin feedback loop at a subset of IES borders.

### Coi6p Interacts with Coi7p and Lia5p

**Figure 4:**
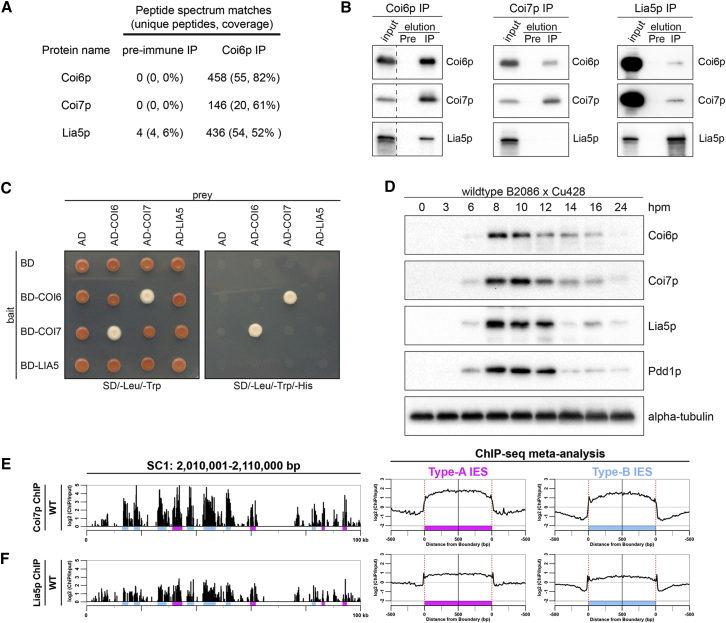
Coi6p Interacts with Coi7p and Lia5p (A) The numbers of peptide spectrum matches, unique peptides, and protein coverage identified by a mass spectrometry analysis of the proteins that precipitated with the anti-Coi6p antibody or with a pre-immune serum from WT cells at 8 hpm. (B) The cell lysate (input) and proteins that precipitated with the indicated antibodies (IP) or with the corresponding pre-immune sera (Pre) from WT cells at 8 hpm were analyzed by western blot. (C) Yeast two-hybrid assay. Yeast strains expressing the Gal4 binding domain (BD) fused to Coi6p, Coi7p, or Lia5p (bait) were mated to strains expressing the Gal4 activation domain (AD) linked to Coi6p, Coi7p, or Lia5p (prey). As controls, strains carrying the empty bait and the prey plasmids were used. Cells were plated on a control plate containing all of the auxotrophic requirements (left) and on a test plate without histidine (right). (D) The expression of the indicated proteins in WT cells at the indicated time points of conjugation was analyzed by western blot. (E and F) The chromosomal localizations of Coi7p (E) and Lia5p (F) in WT cells at 12 hpm were analyzed by ChIP-seq using the anti-Coi7p and anti-Lia5p antibodies, respectively. The data were analyzed as in Figure 1E. Type-A and type-B IESs are marked in magenta and light blue, respectively. See also Figure S2.

To better understand the function of Coi6p, we aimed to identify Coi6p-binding proteins. Immunoprecipitation (IP) was performed in the lysate of WT cells at 8 hpm using the anti-Coi6p antibody. Mass spectrometry analyses detected Coi7p and Lia5p as co-precipitated proteins with Coi6p (Figure 4A). Coi7p is an acidic leucine-rich nuclear phosphoprotein 32 (ANP32) family protein (Figure S2C) that is encoded by the conjugation-induced gene *COI7* (Woehrer et al., 2015). Lia5p is similar to IS4 family transposases but probably lacks endonuclease activity (Shieh and Chalker, 2013).

**Figure 5:**
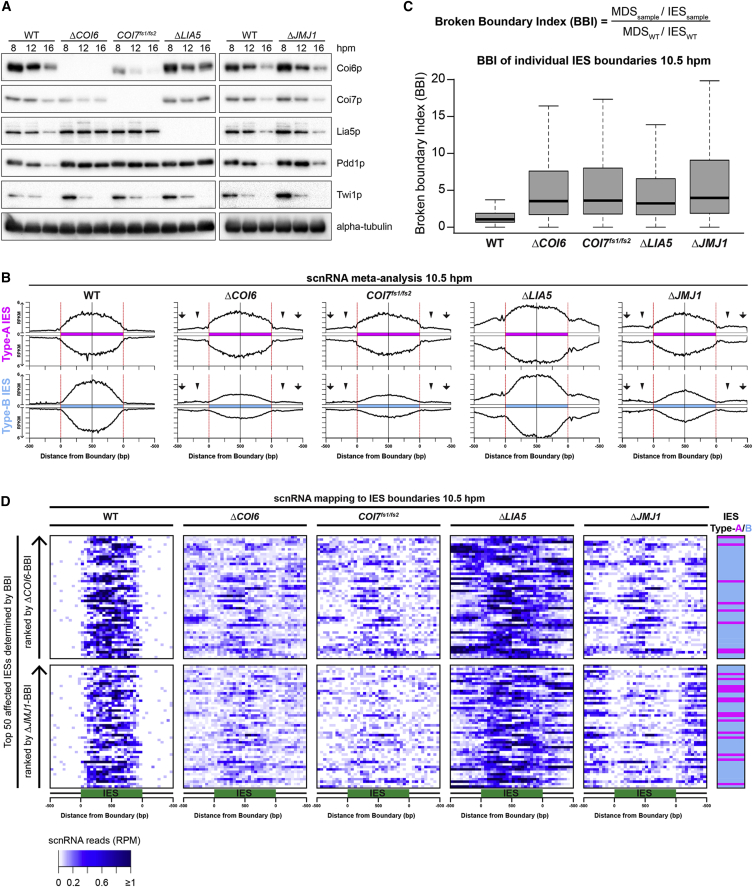
Coi6p-Interacting Proteins and Jmj1p Confine Late-scnRNA Production within IESs (A) The expression of the proteins (indicated at right) in the strains (indicated on top) at late conjugation stages (8, 12, and 16 hpm) was analyzed by western blotting. (B) Small RNAs from the indicated strains at 10.5 hpm were sequenced, and 26- to 32-nt RNAs (scnRNAs) were mapped to compiled type-A (top) and type-B (bottom) IES loci as in Figure 2I. Arrows and arrowheads indicate MDS regions distal and proximal to IESs, respectively, which were differently affected in *ΔCOI6*/*COI7*^*fs1/fs2*^ cells and in *ΔJMJ1* cells. (C and D) Small RNAs from the indicated strains at 10.5 hpm were analyzed as in (B), but for individual IES boundaries. At each boundary, the number of reads mapped to 500 bp outside of the boundary (MDS_sample_) was divided by the number of those mapped to 500 bp inside of the boundary (IES_sample_). Then, the same calculation was performed with small RNAs from control WT cells at 10.5 hpm (MDS_wt_/IES_wt_), and the broken boundary index (BBI = [MDS_sample_/IES_sample_]/[MDS_wt_/IES_wt_]) was calculated. The BBIs of each strain are shown as a boxplot (C). The top 50 IESs affected (i.e., highest BBIs) in *ΔCOI6* (top) or *ΔJMJ1* (bottom) cells were chosen, and the normalized (read per million reads [RPM]) read number in each 50-bp bin was visualized as a heatmap (D). See also Figure S3.

We raised antibodies against Coi7p and Lia5p whose specificities were verified using *COI7* mutant (see below) and *LIA5* KO (*ΔLIA5*) (Shieh and Chalker, 2013) cells, respectively (see Figure 5A). Reciprocal IP using these antibodies (Figure 4B) showed that Coi7p and Lia5p co-precipitated with Coi6p, whereas only Coi6p co-precipitated with Coi7p, and Coi6p and Coi7p co-precipitated with Lia5p, albeit in low amounts. A yeast two-hybrid assay showed that Coi6p binds to Coi7p, whereas the interaction between Lia5p and the other two proteins was undetectable (Figure 4C). These results indicate that Coi6p interacts directly with Coi7p and probably indirectly with Lia5p.

Consistent with the late conjugation-specific expression of *COI7* and *LIA5* mRNAs (Figure S1C), both Coi7p and Lia5p were detected specifically at late conjugation stages by western blot (Figure 4D). ChIP-seq analyses using the anti-Coi7p or anti-Lia5p antibodies showed that, in the new MACs of WT cells at 12 hpm, these proteins were enriched on both type-A and type-B IESs (Figures 4E and 4F). Therefore, Coi7p and Lia5p co-localize with Coi6p on IESs, and thus these three proteins not only interact in cell lysate, but also likely on chromatin.

### Coi7p Is Necessary for the Stable Accumulation of Coi6p

Even though Δ*LIA5* strains have been described (Shieh and Chalker, 2013), no loss-of-function mutant of *COI7* has been established. By expressing Cas9 and a guide RNA (gRNA) targeting the *COI7* open reading frame (ORF) in WT cells, we produced two heterozygous *COI7* mutants with different 1-bp deletions in the 14th codon of the *COI7* ORF in the MIC, named *COI7*^*fs1*^ and *COI7*^*fs2*^ (Figures S2A–S2D). Then, these heterozygous mutants were interbred to generate transheterozygous *COI7*^*fs1/fs2*^ strains in which all copies of *COI7* in both MIC and MAC were disrupted (Figure S2A). Coi7p was undetectable in *COI7*^*fs1/fs2*^ cells (Figures 5A and S2E), confirming the complete disruption of *COI7* in these cells.

We then investigated whether Coi6p, Coi7p, and Lia5p influence each other’s stability and localization. Western blot analysis showed that, although the absence of Coi6p or Lia5p did not obviously affect the accumulation of the other proteins, in *COI7*^*fs1/fs2*^ cells, only a small amount of Coi6p accumulated at 8 hpm and became undetectable at later stages (Figure 5A). Immunofluorescent stainings showed that none of them were required for the other proteins to localize into the new MAC, although Coi6p was greatly reduced in *COI7*^*fs1/fs2*^ cells (Figures S2F–S2H). Therefore, Coi7p is important for the accumulation of Coi6p but is not required for the nuclear localization of Coi6p.

### Coi7p and Lia5p Are Important for the Precise Production of Late-scnRNAs

We next asked whether any of the Coi6p-binding proteins play a role in Late-scnRNA accumulation. An analysis of small RNAs from *COI7*^*fs1/fs2*^ cells at 10.5 hpm showed that Late-scnRNAs from MDSs were increased at both type-A and type-B IES loci, and that the overall production of Late-scnRNAs from type-B IESs was reduced (Figure 5B). These defects were similar to those of *ΔCOI6* cells (Figure 5B) and can be explained by the destabilization of Coi6p in the absence of Coi7p (Figure 5A). In contrast, in *ΔLIA5* cells, the number of mapped Late-scnRNAs increased on both MDSs and IESs (Figure 5B). Therefore, Lia5p negatively regulates Late-scnRNA production in both IESs and MDSs, whereas Coi6p and Coi7p prevent Late-scnRNA production specifically outside of IESs.

We next determined which fractions of IES boundaries are affected by Late-scnRNA production in each mutant (Figure 5C). For each boundary, scnRNA reads from 10.5 hpm mapping to the 500-bp MDS region outside of each boundary were divided by those mapping to the 500-bp IES region inside the respective boundary, and this value was normalized by dividing it by the corresponding values from control WT cells at 10.5 hpm to obtain the broken boundary index (BBI). Then, distributions of BBIs were visualized with boxplots. Whereas BBIs in WT cells (biological replicate for the control WT cells) at 10.5 hpm centered at approximately 1 (= no disturbance), the distribution of BBIs shifted to higher values in all of the mutant strains, indicating that a large subset, but not all, of the IES boundaries lost the precision of Late-scnRNA production in the absence of Coi6p, Coi7p, or Lia5p.

To compare the Late-scnRNA production of the different mutants at the single IES locus level, we mapped sequenced scnRNAs of each strain at 10.5 hpm to the 500 bp inside and outside of each IES boundary, and the top 50 affected (highest BBI) loci in *ΔCOI6* cells were chosen to show as heatmaps (Figure 5D, top). The heatmap patterns were comparable between *ΔCOI6* and *COI7*^*fs1/fs2*^ cells, indicating that a similar set of IESs was affected to similar extents in terms of Late-scnRNA production in *ΔCOI6* and *COI7*^*fs1/fs2*^ cells. Most IESs affected in *ΔCOI6* cells were also affected in *ΔLIA5* cells, although scnRNAs from individual loci were generally increased in *ΔLIA5* cells. Collectively, we conclude that Coi6p, Coi7p, and Lia5p are crucial for the precise production of Late-scnRNAs from largely overlapping sets of IESs.

### Jmj1p May Cooperate with Coi6p to Regulate the RNAi-Heterochromatin Feedback Loop

The H3K9/K27 methyltransferase Ezl1p is required for the production of Late-scnRNAs (Noto et al., 2015). Because the H3K27 demethylase Jmj1p (Chung and Yao, 2012) potentially counteracts Ezl1p, we hypothesized that Coi6p and its binding proteins cooperate with Jmj1p. *JMJ1* KO (*ΔJMJ1*) cells were established by disrupting all copies of *JMJ1* in both MIC and MAC (Figure S3A). Accumulations of Coi6p, Coi7p, and Lia5p were not obviously affected in *ΔJMJ1* cells (Figure 5A). In agreement with the previous ChIP-PCR analysis of H3K27me3 in *JMJ1* RNAi-knockdown cells (Chung and Yao, 2012), ChIP-seq analyses of *ΔJMJ1* cells showed that H3K27me3 and H3K9me3 were less enriched on both types of IESs than in WT cells and redistributed to MDS regions (Figures S3F–S3I). Coi6p was redistributed similarly to H3K9/K27me3 (Figure S3C), but Pdd1p was detected only at a subset of loci at which H3K9/K27me3 was upregulated (Figure S3E) in *ΔJMJ1* cells. Therefore, similar to *ΔCOI6* cells (Figure 2), H3K9/K27me3 are redistributed, and some mechanism besides these histone marks helps to confine Pdd1p localization to IESs in the absence of Jmj1p.

We then examined small RNAs in Δ*JMJ1* cells and found that Late-scnRNAs were produced ectopically at the loci where ectopic heterochromatic histone marks were also detected (Figure S3K, arrowheads). Late-scnRNA production was similarly affected in *ΔJMJ1* and *ΔCOI6* cells genome-wide (Figure 5B), as well as at the individual locus level for both the 50 most affected IES loci in Δ*COI6* cells (Figure 5D, top) and those in Δ*JMJ1* cells (Figure 5D, bottom). However, in Δ*JMJ1* cells, Late-scnRNA production from MDSs that were distant from IESs was more strongly affected than in *ΔCOI6* cells (compare regions marked with arrowheads and arrows in Figure 5B), suggesting that Jmj1p may act at MDS regions regardless of their distance from IESs. The results above suggest that Jmj1p and Coi6p confine heterochromatin formation and Late-scnRNA production to IESs at a largely overlapping set of IES loci.

### Coi6p, Coi7p, Lia5p, and Jmj1p Are Important for DNA Elimination

**Figure 6:**
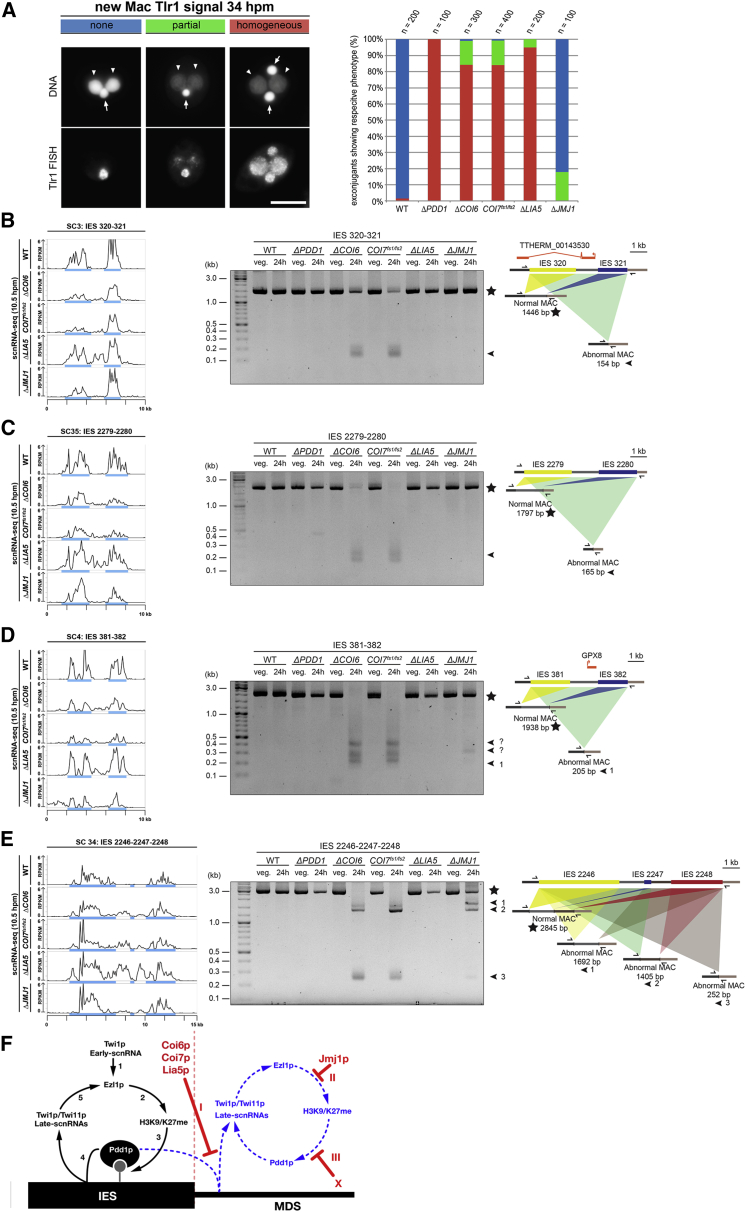
Disturbed Late-scnRNA Production Is Associated with Abnormal DNA Elimination (A) Cells were fixed at 34 hpm and hybridized with fluorescently labeled probes complementary to the moderately repeated Tlr1 IES elements. DNA was counterstained with DAPI. Exconjugants were categorized into three classes (left): (1) DNA elimination was completed, and thus the FISH signal was absent in the new MACs (arrowheads), but present only in the MIC (arrow) (blue); (2) DNA elimination was partially inhibited, and thus the FISH signal was inhomogeneously dispersed in the new MACs (green); and (3) DNA elimination was completely inhibited, and thus the FISH signal was homogeneously distributed in the new MACs (red). Scale bar, 10 μm. Exconjugants from the indicated strains in each of the categories at 34 hpm were counted (right). (B–E) Left: profiles of scnRNA-seq at SC3 (B), SC35 (C), SC4 (D), and SC34 (E) MIC loci in the indicated strains at 10.5 hpm. IESs are marked in blue. Middle: results of DNA elimination analyses by PCR using the primers indicated with arrows in the right schematic drawings. For each strain, vegetative cells (before conjugation) and cells at 24 hpm (exconjugants) were analyzed. Right: schematic representations of the MIC loci (top) and the new MAC loci (bottom) with normal DNA elimination (“Normal MAC”) or with expected ectopic DNA elimination (“Abnormal MAC”). Predicted genes are indicated in orange. (F) A model for the roles of the boundary-protecting factors. See text for details. See also Figure S4.

To compare the efficiency of DNA elimination in the different mutants, we harvested cells at 34 hpm and hybridized them with probes complementary to the moderately repeated Tlr1 IESs (Wuitschick et al., 2002) (Figure 6A). DNA elimination is completed at ∼16 hpm in WT cells but is completely blocked in the absence of Pdd1p (Coyne et al., 1999). Consistently, we detected no Tlr1 staining in the new MACs of WT cells and a homogeneous Tlr1 signal in the new MAC from *ΔPDD1* cells. As we reported previously (Woehrer et al., 2015), most of the *ΔCOI6* cells (84%) showed a homogeneous Tlr1 signal in the new MAC, but a significant population (15%) showed a fainter and non-homogeneous hybridization signal in the new MAC (“partial” in Figure 6A), and a small fraction (1%) of the cells even showed no detectable Tlr1 IESs in the new MAC. Similarly, in *COI7*^*fs1/fs2*^ cells, 84%, 15%, and 1% of cells showed homogeneous, partial, and no detectable Tlr1 signal in the new MACs, respectively. In *ΔLIA5* cells, 95% and 5% of cells showed a homogeneous and partial Tlr1 signal in new MACs, respectively. In contrast, in Δ*JMJ1* cells, the Tlr1 signal in the new MAC was detected only in 18% of cells. Altogether, we conclude that Coi6p, Coi7p, Lia5p, and Jmj1p are important for completing DNA elimination, whereas the phenotypic divergence between *ΔCOI6*/*COI7*^*fs1/fs2*^/*ΔLIA5* cells and *ΔJMJ1* cells indicates that Jmj1p has distinct functions from the rest of the proteins in DNA elimination.

### Abnormal DNA Elimination Occurs at the Site of Ectopic Late-scnRNA Production

DNA elimination was severely, but not completely, inhibited in *ΔCOI6*, *COI7*^*fs1/fs2*^, and *ΔLIA5* cells, and was only mildly inhibited in *ΔJMJ1* cells (Figure 6A). Therefore, at least some level of DNA elimination occurred in these mutants. Using these strains, we asked whether the ectopic production of Late-scnRNAs causes ectopic DNA elimination. We chose four genomic loci, each of which continuously produces scnRNAs from MDS regions between two or three consecutive IESs in *ΔCOI6* cells and determined whether these MDS regions were eliminated together with the neighboring IESs. Primers were designed in MDS regions close the “left” boundary of the “left” IES and close to the “right” boundary of the “right” IES (Figures 6B–6E, right panels, arrows) and were used for PCR with genomic DNA from vegetative cells (before conjugation) and from exconjugants at 24 hpm. In this experimental design, we detected only the MAC loci, but the longer MIC loci were inefficiently amplified. Because not all cells in a culture complete conjugation, the detected MAC loci at 24 hpm could be derived from both the new MAC and the parental MAC.

From WT and Δ*PDD1* cells, we detected only the normal MAC loci (Figures 6B–6E, “Normal MAC,” stars) for all of the tested loci. In contrast, from *ΔCOI6* and *COI7*^*fs1/fs2*^ cells, we detected PCR products corresponding to DNA shorter than the normally rearranged MAC loci in all of the loci (Figures 6B–6E, “Abnormal MAC,” arrowheads). The DNA sequencing of the PCR products from Δ*COI6* cells indicated that these indeed lacked the MDS between the IESs (Figure S4). In Δ*JMJ1* cells, similar ectopic DNA elimination was detected at two (Figures 6D and 6E) of the four loci, which correlated with the incidence of ectopic Late-scnRNA production at these loci (Figures 6B–6E, left panels). In *ΔLIA5* cells, we did not detect any such abnormal DNA elimination, likely because of the more severe block of DNA elimination than in the other mutants (Figure 6A). Because the ectopic DNA elimination at two of the loci above resulted in the deletion of genes (Figures 6B and 6D) and because there are ∼10,000 IESs in the MIC genome, many genes could be removed by ectopic DNA elimination in the absence of Coi6p, Coi7p, or Jmj1p. Altogether, the results above suggest that the precise production of Late-scnRNAs from IESs is important for generating a functional somatic genome.

## Discussion

In this study, we demonstrated that the HP1-like protein Coi6p, Coi6p-interacting proteins Coi7p and Lia5p, and the H3K27 demethylase Jmj1p are crucial for many IESs to confine the production of Late-scnRNAs and the formation of heterochromatin to IESs. The presence of these “boundary-protecting factors” indicates the existence of active mechanisms that define the borders of IESs at the level of heterochromatin in *Tetrahymena* by counteracting the positive feedback loop between RNAi and heterochromatin, thus preventing the redistribution of the RNAi signal and heterochromatin into neighboring genomic regions. Because the loss of Coi6p, Coi7p, or Jmj1p caused abnormal DNA elimination, the proper formation of heterochromatin boundaries is required for the integrity of the somatic genome.

Previous studies (Liu et al., 2007, Noto et al., 2015, Taverna et al., 2002) have indicated that the RNAi-heterochromatin positive feedback loop in DNA elimination consists of the following steps (Figure 6F, left): (1) the Early-scnRNA-Twi1p complex recruits the histone methyltransferase Ezl1p to IESs in the new MAC; (2) Ezl1p catalyzes H3K9me3 and H3K27me3; (3) H3K9/K27me recruits the HP1-like protein Pdd1p; (4) Pdd1p induces the biogenesis of Late-scnRNAs in *cis*; and (5) Late-scnRNA-Twi1p/Twi11p complexes further recruit Ezl1p. In this study, we found that, at some IES borders, the loss of the boundary-protecting factors caused the redistribution of Late-scnRNA and H3K9/K27me3 without a severe redistribution of Pdd1p (Figures 2 and S3), despite the necessity of Pdd1p for the accumulation of Late-scnRNAs (Noto et al., 2015). This observation indicates that Pdd1p can act at long distances beyond the IES borders to induce Late-scnRNA production, which we suggest to be inhibited at three different levels in WT cells (Figure 6F, right): (1) at Late-scnRNA biogenesis, in which Coi6p and its associated factors are possibly involved; (2) at H3K9/K27me3 accumulation, at which Jmj1p may act by turning over H3K9/K27me3 either genome-wide or specifically at IES boundaries; and (3) at Pdd1p accumulation, at which a yet unknown factor prevents Pdd1p from being localized to MDSs even in the advent of H3K9/K27me3 redistribution in the absence of the boundary-protecting factors.

Coi6p and its binding partners were distributed across the whole body of IESs according to our ChIP-seq analyses (Figures 1E, 4E, and 4F). Therefore, it is puzzling that they have a specialized function at IES boundaries. Although the loss of Coi6p or Coi7p resulted in abnormal DNA elimination (Figures 6B–6E), it also severely blocked DNA elimination (Figure 6A). The latter is probably not simply caused by the disturbance of heterochromatin borders because although we observed a similar degree of heterochromatin and Late-scnRNA spreading in Δ*COI6* and Δ*JMJ1* cells, DNA elimination was only mildly inhibited in Δ*JMJ1* cells (Figure 6A). Coi6p and its binding partners may play a dual role in promoting DNA elimination at the body of IESs and inhibiting heterochromatin spreading at the borders. In *S. pombe*, Swi6 resides in the bodies of heterochromatin domains and recruits the JmjC domain-containing protein Epe1. Loss of Swi6 or Epe1 causes spreading of H3K9me (Stunnenberg et al., 2015, Zofall and Grewal, 2006). Therefore, two opposing chromatin-modifying activities both reside in heterochromatin to confine heterochromatin at proper loci in fission yeast. Our study suggests that the two HP1-like proteins Pdd1p and Coi6p are recruited to IESs in *Tetrahymena* and have opposing activities for heterochromatin assembly. Like Epe1, Jmj1p is a JmjC domain-containing protein. Jmj1p might be recruited to heterochromatin by Coi6p to negatively regulate heterochromatin spreading.

Coi7p belongs to the ANP32 protein family (Figure S2C), some members of which have been implicated as histone chaperones (Reilly et al., 2014). Coi7p might also be a histone chaperone that regulates histone dynamics. Alternatively, because Coi7p directly binds to and stabilizes Coi6p (Figures 4 and 5A), it might be a “Coi6p chaperone” that controls the localization of Coi6p. Compared with the Coi6p-Coi7p interaction, the association of Lia5p with Coi6p was less robust (Figures 4A–4C). Consistently, the phenotype of Δ*LIA5* cells diverged from that of Δ*COI6* and *COI7*^*fs1/fs2*^ cells with a more severe DNA elimination block (Figure 6A), no detectable ectopic DNA elimination (Figures 6B–6E), and Late-scnRNA upregulation from both MDSs and IESs (Figure 5B). Lia5p probably inhibits the RNAi-heterochromatin feedback loop at both the bodies and the borders of IESs, and the Coi6p-Coi7p complex may enhance its activity at the borders.

In terms of spreading Late-scnRNAs and heterochromatin, not all IESs were affected in the absence of the boundary-protecting factors, and even the two boundaries of each IES seemed to act independently (Figures 2 and S3). We so far have failed to identify any features explaining the vulnerability of each boundary to the spreading event induced by the loss of the boundary-protecting factors. A few IESs are known to be associated with *cis*-acting elements that influence their excision boundaries (Carle et al., 2016, Chalker et al., 1999, Godiska et al., 1993). The heterochromatin borders of distinct sets of IES boundaries might also be defined by different sets of *cis*-acting elements, and the identified boundary protecting factors may be functionally associated with only a subset of such elements. If this is the case, IESs that are not affected by the loss of identified factors may be regulated by another mechanism. Several HP1-like proteins in addition to Pdd1p and Coi6p are encoded in the *Tetrahymena* genome, and some of them might have a boundary-protecting function similar to Coi6p, but at different sets of IES boundaries.

## Experimental Procedures

### *Tetrahymena* Strains and Culture Conditions

The WT strains B2086 and CU428 were obtained from the *Tetrahymena* Stock Center. Δ*COI6* and Δ*LIA5* strains were described previously (Shieh and Chalker, 2013, Woehrer et al., 2015). Other strains are described below. Cells were grown at 30°C in super proteose peptone (SPP) medium (Gorovsky et al., 1975) to a concentration of ∼5 × 10^5^ cells/mL, washed with 10 mM Tris (pH 7.5), starved for 8–24 hr at 30°C, and mating was induced by mixing equal numbers of cells with different mating types at 30°C.

### Antibodies

Rabbit anti-Coi6p, anti-Coi7p, and anti-Lia5p antibodies were raised against respective full-length recombinant proteins and purified with Protein A. Guinea pig anti-Pdd1p antibody was described previously (Kataoka and Mochizuki, 2015). Rabbit anti-Pdd1p antibody (ab5338) and mouse anti-alpha-tubulin antibody 12G10 were obtained from Abcam and Developmental Studies Hybridoma Bank, respectively.

### DNA Elimination Assays

Fluorescence in situ hybridization (FISH) was performed as previously described (Kataoka and Mochizuki, 2015). For PCR analyses, total genomic DNA and the primers listed in Table S1 were used. Products were purified from the gel, cloned, and >20 clones for each product were sequenced.

### ChIP-Seq and Small RNA-Seq

ChIP-seq was performed as previously described (Kataoka and Mochizuki, 2015). The reads were mapped on individual genomic loci with 100-bp bins or compiled 500 bp inside and outside of each boundary of 3,715 type-A and 2,863 type-B IESs with 10-bp bins. The number of ChIP-seq reads was divided by that of input reads. Small RNA-seq was performed as previously described (Noto et al., 2015). The position of the 5′ end of each RNA read was mapped to 100-kb genomic loci (bin size, 100 bp). For the meta-analysis, which compiled results for 500 bp inside and outside of each boundary of 3,722 type-A and 2,863 type-B IESs, and for the heatmap visualization of scnRNA expression, the position of the 13th nt of each scnRNA was mapped in 10-bp or 50-bp bins, respectively. To obtain the BBI, the number of RNA reads that mapped the 500-bp MDS region outside each boundary was divided by the number used to map the 500-bp IES region inside the boundary, and this value was normalized by dividing by the corresponding values obtained from WT cells.

### Identification of Coi6p-Associated Proteins

WT cells were harvested at 8 hpm and suspended in 1× IP buffer (20 mM Tris [pH 7.5], 100 mM NaCl, 2 mM MgCl_2_), including 2× Protease Inhibit Cocktail (without EDTA) (Roche) and 0.5 mM PMSF. The cells were lysed by sonication, PMSF was further added to 1 mM, and insoluble material was removed by a 20 min centrifugation at 17,000 × *g* and stored at −80°C. The lysate from 5 × 10^7^ cells was incubated with 250 μL of Dynabeads Protein A (Invitrogen) cross-linked with either pre-immune serum or the anti-Coi6p antibody for 2 hr and then washed 3× for 5 min with 10 mL and 6× for 1 min with 1 mL of IP buffer. Proteins were eluted in 100 μL of 0.1 M glycine HCl (pH 2) for 10 min at 25°C, neutralized by adding 10 μL of 1 M Tris (pH 9), trypsin-digested, and analyzed by mass spectrometry.

### Protein-Protein Interaction Analyses

For coIP, cell lysate was prepared as above with modified IP buffer (50 mM Tris [pH 7.5]; 100 mM NaCl; 20 mM EDTA; 0.1% Tween 20), 1 mM PMSF, and 3× Ultra Protease Inhibitor Cocktail (Roche). In addition, 1 mM PMSF was further supplemented after thawing the lysate. Then, 1 mL of cell lysate was incubated with Dynabeads cross-linked with immune or pre-immune serum for 2 hr at 4°C and washed 4× for 5 min with 1 mL of IP buffer. The proteins were eluted in 35 μL of 0.1 M glycine (pH 2) for 10 min at 25°C, and 1/10 vol of 0.75 M Tris (pH 9) and 1.25 M NaCl were added. Procedures for yeast two-hybrid assays are in the Supplemental Information.

### Production of COI7 Mutant and *JMJ1* KO Strains

pBNMB1-HA-Cas9Tti-U6gRNA-COI7T1 (see Supplementary Information) was digested with XhoI and introduced to the MAC *BTU1* locus of CU428 cells using a biolistic gun. Transformants were assorted until they grew in 10 mg/mL paromomycin, Cas9 expression was induced in 1 μg/mL CdCl_2_ for 5 hr, and the cells were washed and incubated overnight in 10 mM Tris (pH 7.5). Then, the cells were mated with B2086 strain, single mating pairs were isolated, and progeny were selected in 15 μg/mL 6-methylpurine. The *COI7* locus was amplified by PCR and sequenced to identify mutations. Two heterozygous strains were crossed to generate transheterozygous *COI7* mutant strains. The *JMJ1* KO construct was made by fusing 1.3 kb of the 5′ flanking region, a *neo3* cassette, and 1.3 kb of the 3′ flanking region. *T*he *JMJ1* coding sequence from both MIC and MAC were removed by standard genetic manipulations. See Table S1 for PCR primers.

## Author Contributions

J.H.S., T.N., K.K., S.G., and K.M. performed the experiments. J.H.S., Y.L., and K.M. designed the experiments and wrote the paper.

## Accession Numbers

The accession number for the sequencing data reported in this paper is GEO: GSE87015 (https://www.ncbi.nlm.nih.gov/geo/).

## Supplemental Information

Document S1. Supplemental Experimental Procedures, Figures S1–S4, and Table S1

Document S2. Article plus Supplemental Information
