# Supplementary material for: Negative Regulators of an RNAi-Heterochromatin Positive Feedback Loop Safeguard Somatic Genome Integrity in Tetrahymena
Source: Cell Rep. 2017 Mar 7;18(10):2494–507. doi: 10.1016/j.celrep.2017.02.024 (PMC5357732; doi:10.1016/j.celrep.2017.02.024)
Supplement: Document S1. Supplemental Experimental Procedures, Figures S1–S4, and Table S1 [file mmc1.pdf]

**Cell Reports, Volume *18***

## Supplemental Information

# Negative Regulators of an RNAi-Heterochromatin Positive Feedback Loop Safeguard Somatic Genome Integrity in *Tetrahymena*

**Jan H. Suhren, Tomoko Noto, Kensuke Kataoka, Shan Gao, Yifan Liu, and Kazufumi Mochizuki**

## Supplemental Experimental Procedures

### Western blot and Immunofluorescence staining

For the western blot, the primary antibodies were diluted 1:2000-5000 and were detected by respective secondary HRP-coupled antibody (Jackson ImmunoResearch Lab) diluted 1:10000. For immunofluorescence staining, cells were fixed as previously described (Loidl and Scherthan, 2004). For Coi7p localization, cells were fixed by Schaudinn's fixative (Song et al., 2007). The fixed cells were incubated with the primary antibodies diluted 1:2000-5000 and then with the respective Alexa dye-coupled secondary antibodies (Invitrogen) diluted 1:1000.

### Cross-linking of antibodies to protein A beads

All of the steps were carried out at RT. 50 µl of Dynabeads protein A (Invitrogen) were washed twice with IP buffer (20 mM Tris, pH 7.5; 100 mM NaCl; 2 mM $MgCl_2$; 2 mM $CaCl_2$; 0.1% Tween-20) and then incubated for 30 min with 200 µl of IP buffer and 30 µl of immune or pre-immune serum. For westernblot analysis of Coi7p or Lia5p IP, 150 µl of Dynabeads were cross-linked to 90 µl of anti-Coi7p rabbit serum. Unbound antibody was removed by 3 washes of 3 min each with 1 ml of IP buffer. Beads were equilibrated with 3 washes of 1 min each with 500 µl of 0.2 M Na-borate (pH 9.2) and then incubated with 1 ml of freshly prepared 20 mM dimethyl pimelimidate dihydrochloride (Sigma) in 0.2 M Na-borate (pH 9.2) for 30 min. The reaction was stopped by 2 washes of 10 min with 500 µl of 0.2 M Tris-HCl (pH 8), followed by 2 washes of 3 min with IP buffer. Non-cross-linked antibody was removed by 2 washes of 1 min with 500 µl of 0.1 M glycine (pH 2). Beads were washed (3x 5 min) and stored at 4 °C in IP buffer and used within 3 days.

### Yeast Two-Hybrid Assays

Yeast strains were grown in YPD or SD selective medium at 30ºC. Codon-optimized cDNAs for *COI6*, *COI7*, and *LIA5* (see below) were cloned into pOAD and pOBD vectors (Miller and Stagljar, 2004), which are designed to express fusion proteins with the activation domain and DNA-binding domain of the Gal4 transcription factor, respectively. The pOAD- and pOBD-based vectors were introduced into yeast strains PJ694A and PJ694α, respectively. The transformed haploids were mated, and the interaction between two fusion proteins was analyzed by detecting cell growth on SD selective medium without histidine and with 25 mM 3-Amino-1,2,4-triazole (3-AT).

### Sequence of pBNMB1-HA-Cas9Tti-U6gRNA-COI7T1

> pBNMB1-HA-Cas9Tti-U6gRNA-COI7T1
GTGGCACTTTTCGGGGAAATGTGCGCGGAACCCCTATTTGTTTATTTTTCTAAATACATTCAAATAT
GTATCCGCTCATGAGACAATAACCCTGATAAATGCTTCAATAATATTGAAAAAGGAAGAGTATGA
GTATTCAACATTTCCGTGTCGCCCTTATTCCCTTTTTTGCGGCATTTTGCCTTCCTGTTTTTGCTCAC
CCAGAAACGCTGGTGAAAGTAAAAGATGCTGAAGATCAGTTGGGTGCACGAGTGGGTTACATCGA
ACTGGATCTCAACAGCGGTAAGATCCTTGAGAGTTTTCGCCCCGAAGAACGTTTTCCAATGATGAG
CACTTTTAAAGTTCTGCTATGTGGCGCGGTATTATCCCGTATTGACGCCGGGCAAGAGCAACTCGG
TCGCCGCATACACTATTCTCAGAATGACTTGGTTGAGTACTCACCAGTCACAGAAAAGCATCTTAC
GGATGGCATGACAGTAAGAGAATTATGCAGTGCTGCCATAACCATGAGTGATAACACTGCGGCCA
ACTTACTTCTGACAACGATCGGAGGACCGAAGGAGCTAACCGCTTTTTTGCACAACATGGGGGAT
CATGTAACTCGCCTTGATCGTTGGGAACCGGAGCTGAATGAAGCCATACCAAACGACGAGCGTGA
CACCACGATGCCTGTAGCAATGGCAACAACGTTGCGCAAACTATTAACTGGCGAACTACTTACTCT
AGCTTCCCGGCAACAATTAATAGACTGGATGGAGGCGGATAAAGTTGCAGGACCACTTCTGCGCT
CGGCCCTTCCGGCTGGCTGGTTTATTGCTGATAAATCTGGAGCCGGTGAGCGTGGGTCTCGCGGTA
TCATTGCAGCACTGGGGCCAGATGGTAAGCCCTCCCGTATCGTAGTTATCTACACGACGGGGAGTC
AGGCAACTATGGATGAACGAAATAGACAGATCGCTGAGATAGGTGCCTCACTGATTAAGCATTGG
TAACTGTCAGACCAAGTTTACTCATATATACTTTAGATTGATTTAAAACTTCATTTTTAATTTAAAA
GGATCTAGGTGAAGATCCTTTTTGATAATCTCATGACCAAAATCCCTTAACGTGAGTTTTCGTTCCA
CTGAGCGTCAGACCCCGTAGAAAAGATCAAAGGATCTTCTTGAGATCCTTTTTTTCTGCGCGTAAT
CTGCTGCTTGCAAACAAAAAAACCACCGCTACCAGCGGTGGTTTGTTTGCCGGATCAAGAGCTACC
AACTCTTTTTCCGAAGGTAACTGGCTTCAGCAGAGCGCAGATACCAAATACTGTCCTTCTAGTGTA
GCCGTAGTTAGGCCACCACTTCAAGAACTCTGTAGCACCGCCTACATACCTCGCTCTGCTAATCCT
GTTACCAGTGGCTGCTGCCAGTGGCGATAAGTCGTGTCTTACCGGGTTGGACTCAAGACGATAGTT
ACCGGATAAGGCGCAGCGGTCGGGCTGAACGGGGGGTTCGTGCACACAGCCCAGCTTGGAGCGA
ACGACCTACACCGAACTGAGATACCTACAGCGTGAGCTATGAGAAAGCGCCACGCTTCCCGAAGG
GAGAAAGGCGGACAGGTATCCGGTAAGCGGCAGGGTCGGAACAGGAGAGCGCACGAGGGAGCTT

CCAGGGGGAAACGCCTGGTATCTTTATAGTCCTGTCGGGTTTCGCCACCTCTGACTTGAGCGTCGA
TTTTTGTGATGCTCGTCAGGGGGGCGGAGCCTATGGAAAAACGCCAGCAACGCGGCCTTTTTACGG
TTCCTGGCCTTTTGCTGGCCTTTTGCTCACATGTTCTTTCCTGCGTTATCCCCTGATTCTGTGGATAA
CCGTATTACCGCCTTTGAGTGAGCTGATACCGCTCGCCGCAGCCGAACGACCGAGCGCAGCGAGT
CAGTGAGCGAGGAAGCGGAAGAGCGCCCAATACGCAAACCGCCTCTCCCCGCGCGTTGGCCGATT
CATTAATGCAGCTGGCACGACAGGTTTCCCGACTGGAAAGCGGGCAGTGAGCGCAACGCAATTAA
TGTGAGTTAGCTCACTCATTAGGCACCCCAGGCTTTACACTTTATGCTTCCGGCTCGTATGTTGTGT
GGAATTGTGAGCGGATAACAATTTCACACAGGAAACAGCTATGACCATGATTACGCCAAGCGCGC
AATTAACCCTCACTAAAGGGAACAAAAGCTGGAGCTCCACCGCGGTGGCGGCCGCTCGAGTCTAG
AGTTGTTTGGATAATTAGATCTCTCTCTTTCTATCGTATTTTGCAATAATAGGTATTAACTTTTATAC
TGATTGTTAGTAGATGCCTTCAAATTTTCTTTTTATTTAAATTCACATGCTATATCTTTTAAAACACT
CCACATTTTATTGTTGCTAACTGTGCTATTGATCTTTAAGTCAATAGCTGCTCATTTTGTTGAACTC
CACAGAGACACTAAATTTGTTTATTTTGATGGATGCTTTATAATTAAAGTTACGTAATCTGCTTGAC
ATTTAGCCAACTATATAAAAAAGATCAAAATGTAGCTTAAATCTCAAAAAATCATCATAATTTACT
ATCAAATTATTAAGAAATTCATATAATCACCACTTTATTGACTTTTATTCATCTTATAGAGTGATAG
TAGAGTTGAGCCAAATTGATACTTGTTTACGTTGTATTATTTTGAAATTTTAAAAAAATGAAAATG
AGAGAAAAATTTATTTAAATTTGAGCTTAGAATCTTTAAGGAAGATCAAAAATGGGCTAACTAAA
TGTTAGAGTACGAAGACTGTTCTTGAAATAAAGTGTCATCCTTTACGAATCAAGTTGCTACTTTAA
TGAATAATAGAATTTGAGGTAGAGCTAAAATGAGAGATATAGTAATGCTATTGGATTATATTTGGT
TTGTATGATGGTTTTTCTTTGGTAAATGAATGATATAAATGAAGAGTGGCAATAAAATTAATTGAA
ATTGAATGAAAAAATGAATAGAAATTAAAGAAGAGTATAATTTTATTTTTGAATTTTATTTAAATT
TTAATGCGTGTATTTATTTGGGTGATGTCGACTATATGTGCAATAGGAATAGGCATTTTTTTAATCT
AGAAGTATTTTATTAAGAAGTAAAAGTTATAAATTTTTTTTTATTGTTAGAAAAGATATTAGAAGG
ATTTCAATTTAAACCAAGTATATTAGATATTAAATAATATTAAATAAAACTAAAGATTTTTATTTA
ATTATCTTAAATATATTCTTATTAATAAAAATAAATCTAAAAACATAGATAGATAAGTAAATAAAT
AAATATATATACTGGTTTAAAATTAGTATTTGTCTTTCAATCAAACAAATACTTGAAAAATACCTA
TTTAAGTTAATGTGTATGGATATTATTTCAAGCTAAGTAATTTATTTTCTATCTAAGAAATAATTCA
AATAGTAAGTTAAAAATTCAGTCATTCAGCTAATTTATAAATAAATCAATCAATCAATAAAACAA
ACAAACAAACAAACAAACAAACAACCATTCGTCTTTTGAATTTTAAAAATTGATTTTTACATCATT
TTATTTAACTTTAAAATAGAAATGAGAGATTAATTGTATTTTTTTAAAAAAAAATTATATTTTTGCAA
TTAGATGTTTGAATTTTTTTCAATGAGTTTTCTAAATTTCTTAATAAGTGGTTTCTTATCAAAAATTA
TAATTAAAAAGGAAACCCATAAAATTAATTATTAAAAATAAATTAGCTTTATAAGATTTCAAAAA
CTTTAATAGTTGAGGCTCTCAAATAAATTAGTgttttagagctaGAAAtagcaagttaaaataaggctagtccgttatcaacttgaa
aaagtggcaccgagtcggtgcTTTTTTTTGTAAATTTTAATTACATAGAAAACAAAAAAGTATTACATTATTTT
AAATAAAAATATTAAATTTTAAATAAAGAATTATTTTCAAATAATTTTCAACTTTTAATAAATAAA
ATTTTAACTTTAATATAAAACAAACTATTTATTTCTAAATTAATTAAATAAATTTGATGTTTATTTG
TTTATTTAGTGATAGACGCAAACTAAATTATTAGTCGACTTGATATCTTCAAAGTATGGATTAATT
ATTTCAAATTATTAGAAGGTAATTAATCTGCATAAATTCAAAACTATAAAAATAAAACATTAAAAT
TAATTCAACCTTATTGAAGCATCAAAATCTGAATCTCTAGAAAGACTGATTCTGATTGGATAATTT
TTCGGCGCTAAGGATTTTGGATTAAAGAAAATTAGATTTAATTATTAATCATGATTTGAATAGGAT
AGCAAGAATATTTGTTTGGTTTAAAAGGGAAAGCGGGTAATTATCAAAAATTTATAAATAATTTTA
AAACAATAAATAGAAAAACAAATAAGATTATAAAAACTTACAAAAATGATTGAACAAGATGGTTT
ACACGCTGGTTCTCCCGCCGCTTGGGTCGAAAGACTTTTCGGTTATGACTGGGCTCAACAAACCAT
CGGTTGCTCTGATGCCGCCGTCTTCCGTCTTTCTGCTCAAGGTCGTCCTGTTCTTTTCGTCAAGACC
GACCTTTCTGGTGCCCTTAATGAACTTCAAGATGAAGCTGCCCGTCTTTCTTGGCTTGCCACCACCG
GTGTTCCTTGCGCTGCTGTCCTTGACGTTGTCACTGAAGCCGGTAGAGACTGGCTTCTTTTAGGTGA
AGTCCCCGGTCGAGATCTTCTTTCTTCTCACCTTGCTCCTGCCGAAAAAGTTTCTATCATGGCTGAT
GCTATGCGTCGTCTTCATACCCTTGATCCCGCTACCTGCCCTTTCGACCACCAAGCCAAACATCGTA
TCGAACGTGCTCGTACTCGTATGGAAGCCGGTCTTGTCGATCAAGATGATCTTGACGAAGAACATC
AAGGTCTTGCCCCTGCCGAACTTTTCGCCAGACTTAAGGCCCGTATGCCCGACGGTGAAGATCTTG
TCGTCACCCATGGTGATGCCTGCTTACCCAATATCATGGTTGAAAATGGTCGTTTTTCTGGTTTCAT
CGACTGTGGTCGTCTTGGTGTCGCCGACCGTTATCAAGATATTGCCTTAGCTACCCGTGATATCGCT
GAAGAACTTGGTGGTGAATGGGCTGACCGTTTCCTTGTCCTTTACGGTATCGCCGCTCCCGATTCTC
AACGTATCGCCTTCTATCGTCTTCTTGACGAATTCTTCTGAGATCCTTAAATTAAAAATTCAATATA
TATTTACAAACTTTCATATAAAATAAATATATTATATAAAATTAATTTTTAGTGTATTATATTAACA
TTAAAGCACCAAAAAAAACGTGTTAATATACTACTATAAAATATAATTTATTCCAAATTGACTAAAA
TCATTATTTTACAACTCATTTGTATATATATATTTTATGTCAATTATTTTTTTTAACTTTCTAAAAAAAA
AAATTCCTCTTCACATACATGTTAGCTCTTAAAAATTTGTCTGCAAATCCAATAATAATATTTTTTT
TTTGCCATTAAATTTTCAAATTTTTACTGGAAAAATGCAGCCCGGGGGATCAGACAATTTATTTCT
AAAAAATATTTAAAAATAAAAAATAATAAGGGTTTTGAATAACTCCTTTAATTTAAATACACATTT

TTAAATTTTTTTAGCTCTTTAAATATTCATAAAAATAAAAAATAACTAACTAAAAATAAATAAAA
AGATAATAATGATTAAAGGTATAATACTGTATAAGAAAAAACATAATAGAGTACTTATTTTTATA
TCACTATTTTAATATCTTGAAAGCAAAACTTTTTATATATCTTAAAATATATTGTATCGTTTATTC
AATTATTTCTTTAAATTTCAAATATATTGATAAAAAAGATGACATGTTTTTAAAGAAAACATGA
AATATAAAATAGATAAATATCAATTATTTTATTTATTAAATATATAAGCTGCTCAAAACATAGCTC
ATTCATCAATTATAATATGTGAATCATTAATTTTCAAAATATTACTCATTATTTAGGCTATCATTTA
TTTTTTATTTTCAATTATCCGTTTCTATTATATTTTAATATTAAGTTGTGATTCTTGAATTTTGTGTCA
TGAATTATTTGTAAATCTTTTTATTTCTGATAAAAAATACAAATTGATTGACTCATGATTTAAATCA
TGAGTCAACCTAACTAATTTTCAAAATTCTTCTATTCTAAAATATAGATGTGATTCTTGAATCTCTC
TTGAATATAAAGTAATTTTTTATATTTCTGATATAATTCTTAGCTACGTGATTCACGATTTATGCAA
TGATCCATATAAAATAATGTAAATAGTGTATATATATATATTCGTCTTTTTTATTCTTTATATAATTT
AAAAAAATTAAAAAAAATTTAATAAAGCTCTAATAAAATAAATAATAATACTAAACTTAAACATAT
GGGATCATATCCTTATGATGTTCCTGATTATGCTGGATCCATGGATAAGAAATATAGCATCGGATT
AGATATTGGTACAAATAGTGTAGGTTGGGCTGTTATAACTGATGAATATAAGGTTCCTAGTAAAAA
GTTCAAAGTTTTAGGTAATACAGATAGACACAGCATTAAGAAGAACTTGATCGGTGCTCTTTTATT
TGATTCAGGTGAAACCGCTGAAGCTACTAGACTTAAACGTACTGCTCGTAGACGTTATACCAGAA
GAAAAAATAGAATCTGTTACCTTCAAGAAATATTTTCTAACGAAATGGCAAAAGTTGACGATTCAT
TTTTTCACAGACTCGAAGAATCTTTTCTTGTTGAAGAAGACAAAAAACACGAACGTCATCCAATCT
TCGGAAATATAGTTGATGAAGTTGCTTACCATGAAAAATACCCAACTATTTATCACTTGAGAAAAA
AGTTAGTTGACTCTACTGATAAGGCTGATCTTAGATTAATTTACTTAGCTCTTGCTCACATGATAAA
ATTTAGAGGACATTTTCTCATTGAAGGAGATTTGAATCCTGACAATTCTGACGTAGATAAGTTATT
CATACAACTTGTCCAAACTTATAATCAATTATTTGAAGAAAATCCTATTAATGCTTCTGGAGTCGA
TGCAAAAGCAATTTTGTCAGCTAGACTCTCAAAATCTAGAAGATTAGAGAACTTAATCGCTCAATT
ACCAGGTGAAAAAAAGAATGGTCTTTTTGGTAATTTGATCGCTCTCTCTTTAGGACTTACTCCTAA
CTTTAAATCAAACTTTGACCTCGCTGAAGATGCTAAGTTACAATTATCAAAGGATACTTATGATGA
TGATCTTGATAATTTATTGGCACAGATCGGTGATCAATACGCCGATTTATTCCTCGCTGCAAAAAA
CTTATCAGATGCTATCTTATTATCTGATATATTAAGAGTTAACACTGAAATTACAAAAGCCCCTTTA
AGTGCAAGTATGATTAAAAGATATGATGAACATCACCAAGATCTTACTTTACTTAAAGCCCTCGTC
AGACAACAATTGCCAGAGAAGTACAAAGAAATATTTTTCGACCAATCTAAAAACGGATATGCAGG
TTACATTGACGGTGGTGCTTCACAGGAAGAATTCTACAAATTCATAAAGCCAATTTTAGAGAAAAT
GGATGGTACTGAAGAATTACTCGTAAAACTTAATAGAGAAGACTTATTACGTAAACAGCGTACAT
TCGATAACGGTAGTATACCTCACCAAATTCATTTAGGTGAACTCCACGCTATCCTCAGACGTCAAG
AGGATTTTTACCCTTTTTTAAAAGATAATAGAGAAAAAATTGAAAAGATACTTACATTTAGAATTC
CATATTATGTTGGTCCTCTCGCTAGAGGAAATTCTAGATTTGCTTGGATGACTAGAAAGAGTGAGG
AGACTATAACTCCCTGGAATTTCGAAGAAGTCGTAGATAAAGGAGCATCTGCTCAATCTTTCATAG
AAAGAATGACTAACTTTGATAAAAATTTACCTAATGAAAAAGTTCTCCCTAAACATTCATTATTGT
ATGAATACTTCACTGTTTACAATGAATTGACAAAAGTTAAATACGTCACTGAAGGAATGAGAAAA
CCAGCTTTTTTGTCTGGTGAACAAAAGAAAGCAATTGTAGATTTATTATTCAAGACTAACAGAAAA
GTTACTGTTAAACAATTAAAAGAAGATTACTTTAAAAAGATCGAATGTTTCGATTCAGTTGAAATA
TCTGGTGTTGAAGATCGTTTTAACGCTTCACTCGGTACTTACCATGATTTGTTAAAGATTATTAAAG
ACAAAGATTTTTTAGATAATGAAGAGAATGAAGACATATTAGAAGATATTGTTTTGACTTTGACTT
TGTTTGAGGATAGAGAAATGATTGAGGAAAGATTAAAAACATATGCTCACTTATTTGACGATAAA
GTCATGAAACAGCTTAAGAGACGTAGATACACTGGTTGGGGAAGATTATCTCGTAAATTGATAAA
TGGAATCAGAGATAAACAAAGCGGAAAAACTATTTTAGACTTCTTGAAGTCAGATGGTTTCGCTA
ATAGAAATTTCATGCAACTTATCCATGATGATTCATTAACATTTAAAGAAGATATACAAAAAGCTC
AAGTCTCAGGTCAAGGAGATTCACTCCATGAACATATTGCTAACTTGGCCGGATCACCAGCTATTA
AGAAAGGTATTTTGCAAACTGTTAAGGTTGTAGATGAACTCGTCAAAGTCATGGGTAGACATAAA
CCTGAAAATATTGTCATTGAAATGGCAAGAGAAAACCAAACAACTCAGAAGGGTCAAAAGAATTC
ACGTGAACGTATGAAAAGAATTGAAGAAGGTATCAAGGAACTTGGTAGCCAAATCTTAAAAGAAC
ACCCTGTCGAAAATACACAACTTCAAAACGAAAAGTTGTACTTATATTATTTACAAAATGGTAGAG
ACATGTACGTAGATCAAGAATTAGATATTAATAGATTGAGCGATTACGATGTAGATCATATCGTTC
CTCAGTCTTTCTTGAAGGATGACAGCATTGACAATAAAGTTTTAACCAGATCTGATAAAAACAGAG
GAAAATCTGATAATGTCCCCTCTGAAGAGGTAGTTAAAAAAATGAAAAATTATTGGAGACAATTA
TTAAATGCCAAACTTATTACTCAACGTAAATTTGATAATTTAACTAAAGCTGAAAGAGGAGGTCTT
TCTGAACTTGATAAGGCAGGTTTCATTAAGCGTCAACTTGTCGAAACACGTCAAATTACAAAGCAT
GTTGCTCAAATTTTAGATAGTAGAATGAATACAAAATATGATGAAAATGATAAATTAATTAGAGA
AGTTAAAGTTATTACTTTAAAAAGCAAACTTGTCTCTGATTTTAGAAAGGATTTCCAATTTTATAA
AGTCAGAGAAATTAATAATTACCATCACGCTCATGATGCTTATTTAAACGCCGTTGTTGGTACAGC
TCTCATTAAAAAGTACCCAAAACTTGAAAGTGAATTTGTTTATGGTGATTACAAAGTCTATGATGT
CAGAAAAATGATTGCTAAGAGTGAACAAGAAATTGGTAAAGCTACAGCTAAGTACTTCTTCTATA

GCAATATCATGAATTTCTTTAAGACCGAAATTACACTCGCTAATGGTGAAATTAGAAAGAGACCTC
TTATTGAGACAAACGGAGAAACTGGTGAGATAGTTTGGGATAAGGGTAGAGATTTTGCCACTGTC
AGAAAAGTTCTTAGTATGCCCCAAGTTAATATTGTTAAAAAAACAGAAGTTCAGACAGGAGGTTT
TTCTAAGGAATCTATCTTACCAAAGAGAAATTCAGATAAGTTAATCGCTAGAAAGAAAGATTGGG
ACCCTAAGAAATATGGAGGTTTTGATAGCCCCACTGTTGCCTACAGTGTTTTAGTAGTTGCTAAGG
TTGAAAAAGGTAAAAGTAAGAAACTTAAATCTGTTAAGGAATTGTTAGGTATTACTATCATGGAA
AGAAGTTCTTTTGAAAAAAACCCTATTGATTTTCTTGAAGCTAAGGGTTATAAAGAAGTCAAAAAG
GATTTAATCATCAAACTTCCTAAGTATAGCCTTTTTGAACTTGAAAATGGACGTAAAAGAATGTTA
GCTTCTGCAGGTGAGCTCCAGAAAGGTAATGAACTCGCATTACCATCTAAGTACGTAAACTTCTTA
TATCTCGCTTCTCATTACGAAAAATTAAAAGGTTCTCCAGAAGATAATGAGCAAAAGCAATTATTC
GTTGAACAGCACAAGCACTATTTGGACGAAATTATAGAACAAATCTCTGAATTTAGTAAAAGAGT
TATATTAGCTGATGCTAATTTAGACAAAGTTTTGAGCGCTTATAACAAACACAGAGATAAGCCAAT
CAGAGAACAAGCTGAAAATATTATCCATCTCTTTACTTTGACAAATCTCGGAGCTCCTGCCGCTTT
CAAATATTTTGATACTACTATTGACAGAAAAAGATATACCTCAACTAAAGAAGTTCTTGATGCCAC
CTTGATACATCAATCTATTACTGGTTTATATGAGACAAGAATCGACTTGTCTCAATTAGGTGGTGA
TGGATCTTCTAAGGGTAAGAAAAAAATCCAAAGAAGGAAAGACTGGAGCTTATGGCAAGAAGGCA
AATAAAAAATAATGAACTAGTTGAGCGAACTGAATCGGTCAGCTAAACCAACCAATCAACATAAT
AAACTTTATTATTTTTACTTAAGCATCTTACTGTTGTTGTAATAGTAGAGAAAGAAATACCCAATTA
ACTTCATTCACATAACATTAATATCTATAAACATCTTTTTTCTCACATATATACAACTCTCTAAATC
AACAAATAACTTTTTAAAAATAATGGATATATATTAACAAAATAATATATCTCTTTTTACAAAATA
GTTCTTATATAAATACGTATTCTGCACTCACCCGCATTTTTCACAACAAAAACATACCAAAAAAAT
TCTTACTTCTACATGTTTCCTTTCTTATTATTACAAAATTATTTTATAAATAGCATACAAAAATAAA
TACAATAAAAAAATAAACAAAATCCTTTTTTATTTTGAATTATTTAAAACAAATATTTTCAATCAA
TCAGTCAGTCAGCATAATATTAAAGCAACAAAACAAACCCAAGTTGTTTTTATAGTTTTTTAATTG
CTTTTCAGTACTATAAATAAATTTGTTATTACTTCAAGATTGATAAACTTCTTTTTTAAATTAAATA
TCTATGAATGAATAAATAAGTTGATATCTCTTTTTAACTTGTTTTCCTCTCTTTTACTTACTTGCCAA
TTTTTTTTTTAAATTAAAGAAATATCTTTTTATTTTTCAAAAACAAAATTTATTTTCCCTTGTATACA
AAAACCCCTTTATTTAAATAAAATCTTTATGCCCATCAATAGCCACATCTTCTCGAGGGGGGGCCC
GGTACCCAATTCGCCCTATAGTGAGTCGTATTACGCGCGCTCACTGGCCGTCGTTTTACAACGTCG
TGACTGGGAAAACCCTGGCGTTACCCAACTTAATCGCCTTGCAGCACATCCCCCTTTCGCCAGCTG
GCGTAATAGCGAAGAGGCCCGCACCGATCGCCCTTCCCAACAGTTGCGCAGCCTGAATGGCGAAT
GGGACGCGCCCTGTAGCGGCGCATTAAGCGCGGCGGGTGTGGTGGTTACGCGCAGCGTGACCGCT
ACACTTGCCAGCGCCCTAGCGCCCGCTCCTTTCGCTTTCTTCCCTTCCTTTCTCGCCACGTTCGCCG
GCTTTCCCCGTCAAGCTCTAAATCGGGGGCTCCCTTTAGGGTTCCGATTTAGTGCTTTACGGCACCT
CGACCCCAAAAAACTTGATTAGGGTGATGGTTCACGTAGTGGGCCATCGCCCTGATAGACGGTTTT
TCGCCCTTTGACGTTGGAGTCCACGTTCTTTAATAGTGGACTCTTGTTCCAAACTGGAACAACACTC
AACCCTATCTCGGTCTATTCTTTTGATTTATAAGGGATTTTGCCGATTTCGGCCTATTGGTTAAAAA
ATGAGCTGATTTAACAAAAATTTAACGCGAATTTTAACAAAATATTAACGCTTACAATTTAG

**Sequences of yeast codon-optimized cDNAs for *COI6*, *COI7*, and *LIA5***
>COI6Sc
TCGAATTCCAGCTGACCACCATGGCCAAGATTAAGTACGAAGGTTCCCAAAAGCAAAACGTCAAG
AAAAGAACCATGCACGTTGTTGATGATGACGAAGATGAAGAACAACCAGTTTACCCAATCGAAGA
AGAATACAACGTCGAATACTTGTACGGTAAGAAGTTCGAAAACGGTCAAATCAAGTACTGCGTTA
AGTGGGAAAACTATACCTTCGAAGAATCCTCATTCGAACCAGTCGAAAACTTGGAAAACGTTGTC
TACAATATGAGAGGTTTCGAAAGAAAGATGTCCGACTTGATTTTCAGAGTTGCCTTGTTGCAAAAC
GCTAAGAAGAAATTGCCACCATTCCAAGTTAACCCAATCCAATTGATCCAAAAGGACAACATCGA
AAACACCAAAGAACAAATCGAACCATCCCAAGCTCAAAACCACTTGCAAAAAGAACCTTCCGTTA
TCAACAATCAAACCTCCGTTGAAAAGTTCCAATCTGCTCCACAACAATCCCCAAAAAAGATTAAC
GGTGGTATCTCCGATAATCAAATCACCCAAAATCAACAAAGTTTGATCGAAACTAACTTGAACTCC
AACAACAACTGCCAAATTCAAGGTTCTCCTAAGTTGGCCGAAAATTTGGAAAAACCACAAAACGG
TTCTCAAAACTTGGCTGAAAAGAACTACTTAAAGACCAACGGTAACTCTAACAATCAATTGCAAC
ACAACCATAACAATCAACAACAAAACAACGGTAATCAATTATTGAACTTGAATCAATCTTTGAATT
TGTTGCAATCCAACGGTCAACACCAATCCCAACAAAAGCAATCTTCTAACGGTAACAATCAAAAC
TCTCAAAACAAGCAATCCTTGTCCTACAACTTGAATGGTAATGGTCAACATCAAAAGAAAGTCGA
AAATCAAGCCATCGGTCAAGTCAACTCTATTTCTCAAGTCCAAAACAAACAAACCCAATTATACG
AAAAGAACAGAAGAACTAACTCCCAAGACCAATATATCCAAACTAAGTTGGACCAAAACTTGTCT
TACATCTCCCAAAAACAATTGCCTAAGCCAATTGAAGATAACAAGACCATGGAAGTCGTCCAAAC
AAAAAACAAGTCCCAATTACAATTCAGAGATCCAGTTTTGGAAAATGAATTGAACTCTAACGCTT
ACTCCGCTCAAGTTGTTGTAGATGATACTTCTAAGTACGGTAACTTCAACAACGGTGATATCCCAT

TGAAGATTTTGAAACATGCTCCATACACCAGAATTCAAAAGGTCATTGGTAACAACGTTGATTTGC
CATCCTCCTTGTACTTCAAGGTTATTTTCAAACCTAGACCATCCGGTACTGTTCCACAACCAGCTTA
TATTGCTTTCAACGAATTGAAGGACAGATACCCAAGAGTCTTGATGGAATATTACGAACAACATG
CCATCTTGTTGGATCCAGTTCCAGATCAAGCTGAATTGCAATTACAAAAGACTAACTCCAGAGACT
TGAACGTTAAGAACGATGCCTCTTTGATTTCTAATGCCGGTAACAACAAGAAGATGGTCAACGGT
AAAGAAAACAGAATCAACATTTGAGCAATTCCCGGGGATCCGTC

>COI7Sc
TCGAATTCCAGCTGACCACCATGGAATTATTGGATTCTGATATCGGTTCCCAAATCAATCAACAAG
TCGAAGGTAAGAACTTCAAAGAAGTCGAAGATATCTACTTGCAAAACATCCAATCTGCCAAGTTC
ACCTCTGAATACAGAGAAAAGATTCAATCCTTCCCAAACTTGATCTGCTTCTCTTTGACTAACATG
AACTTCTCCAAGATCCAAGACTTGCCAATCTTGAAGAGATTATTGAGATTGGAAATCTGCTACTGT
ACCTTCGATATCTCCTCCTTGAATCAAATCTACATCCAATTCCCAAGATTGGTTTCCTTGAGATTAG
TTGGTTGCAACATCTCCTCTTACTCCCAAATTGAATGTTTGACCTCTTTGCCAGATTTGGCCCAATT
GGATTTGTTTAACAACCCAATCTACAAGTTCAACTACGAATACCAATCTTTCCACCAAAAGATGTT
CGAATTATTTCCAAAGTTGATGTACTTGGACAACTTGAAGAAGGATTTCTCTAGAGCTTGTACCAT
CCCTATCAATCAAATCGATCCAAAGCAATTGATCCAAGAAGTTGAAATCCCAAAGTTCGTCGACA
AGTTGAACAGATCCAACATCAACTCTCAAAACAACTCCTTCAACTCCAACAAGTTGACTAGAAAG
AACAATATCAACAACCCTAACAAGAGAAGAGCCGATAACTCTGATGAAGAAGATGACGATTTGGA
CGAAGATTACCAAGAAGATTCTGACGAACCATCCAATCAAAGACATGGTTACCAAACCAGAAAGC
AACAAAAATTGGCTGCCTACAAGTAAGCAATTCCCGGGGATCCGTCTGA

>LIA5_Sc
TCGAATTCCAGCTGACCACCATGGAATTGGGTGAAGCTGACTTGCATACTTCTCAATCTATCGTTC
AAGAAGAAGTTCCAATGCAACAAGAAGTCAACATCCAAGAAGAACAAAACTCCCAATTCGACAA
CGTTCAACAACAAAATCAAAACGTCGAAGAACAACACCAAACTAACAAGAACAACTTGACCCAA
AACGACTTCAACGAATTCCAACAACAAAACACCAACGAACAAGACTTGCCAAGAAACGATCAAA
CTCAAGTTCAACCTCAACAAAACTTGCAAGACGAAAACAATCAAGACCAAACCATCCATTTCACC
GAACAACATTTCCAACAAAGAGAAAGAGAATCCTCCGAAACTCACGATACCTCTAATCAAGAATT
GGAATACCAAATCAATCAAAACATTAACGAAAACAAGCAAGTTCAAAAGGACAACTTGAACGAA
GAAATCATCTCCCAAAACAAAGAAGCTTTGGACGGTCAAGAAGATTACCAAAACTTTAACAAGAA
GGACTTGCCAGACAACAACAATTCTAACGAACAACACAATCAAGATCAAGAAAACATGAATCAA
ATCGAATCCCAAGAAGGTCAAAATCAATTGGTCCAAATCGAAGTTAACGGTTCCGTCAATTCATTG
CAAGAAAACAACTACATCCAAGACGGTACTTTCATGGAACAAGAACATTTGAACGACAAGATGCA
ACAACAACAAGAACAAGCCGTCAAAGAAGAAACCGAAGAAAGAGAAGAAATGCAAGAAGAAAA
TCAATTACACGAAAATCAAGAAAACATCTCCATCTCCCAACAAAATCAACAAGCCCATTCCGATA
TGCAAATTGAAGTTCAAGCCAATCAAATCAATCAACAAGAAAATCAATTGGAATCCTTGAACGAC
AAAGAAGTCGTAAAAGAAGAAAACAACTTGTTATTGAACATCTTGTCCGACGAAAACAACAAAAG
ATTCCAAAGATTGCCACCAGAATTGTCCCCAAACATTTTCTGTCATTTTTTGGGTGGTAAGTCCTTG
CAATTGACCAACATCAATGACTCCTCCAGATTATTCATGTACTTTTTGGGTACTAAGATCTTGAGA
CAATTATTGAACAAGGCCAACGTCAAGTTGAGAGAAATCTACAAAAAGCAAAACGATGTCAAAG
AATACACCATTGAAGAAATCCAAGTCTACCAAGCCTTGAAGATTTTGATGGGTTTACAACAAAACT
CTTCTAACTTCGACTTTTTCTTATTGAAAGACTTGCAAAAGATCCCACCATACCACCAATTATTCAC
TAACGAAAGATTTCAATTCTTGTTGGACTGCGAAAGAGAATTGAACAAGGACATCTTGAACAACG
AACAATTGATCCAAGACTTTGTCCAAAGAGCCCAAAATTCTCAAACTTCCGACCAAGAATTAGTCT
TGATCTCCAAAAAAGGTAAGGTTGGTGAAGAAATTATCCACAACAACTCCGTTTACACCCAAATCT
TTTTGTGCGAATTGTCCTCTGCTTTCGTTTTCGGTTACTTCGTTGTTAAGGACATGTCATCCTTCGCT
AATCAAATCTGCATCAACTTGGAAAGTTTCACTAATCAAAACCACCACGTCTACTTCCAAAACGAA
GAATTTTTCTCCAACTACGAAAAAATTCAAGAATTATTGAACTCCAAGATCCACATCTCCTCATTTT
TGAACAACAAGTTCGACCAATTCCCACAATCCTTACAAAACGAAATGTTGCACAACAAGCCATTG
AAGGCTAACAACTCTGAAACCATCTTCGATAGACAAACCCAAACCCAATTATTGATCAAATCTGA
CGAAAACTTGAAGAAAGAAGTCTTTTTGACCACCTCCGGTACTGTTAAGCAAGATAAGATGGTTG
AAAAGCACAAGCAAGCTATCCAAAAGATCACCCAAAAGTTGAGAATGTTGTTGACCGAATACAGA
TTCGTTTCCATCAACAACGATACCACCTCCATTTTCGAAGAATTGTCTGAAATTGCCATTCAAAATT
CCTACATCATCTACTCCCAAGCCAAAGAAAAGATGGACTATAGATTATTCAGATTCAAGTTGGCCC
AAGACTTGTTGCAAAAGCAAATCCAAAAAATCAAGCAAGAAGAATTGCAAAACGTCAAGTCTAA
GTTGATCGATGTTGAAGTCCAAACCGATAAGGTTGTTGAATCCGTTATTAACGTTGAAACCCATGC
CTTGTCTAAGTCTCCAAAATCCTCTGATAACAATGAACAATTCATCATGAACGAAGTCAACTCCCC
AGTTTTTCAAGGTAACGACCAAAACATAAGACAAGGTGGTACTCATGTTCAAAAGAAGGATGGTA
AGCAAGGTATTTGCTTGGTTTGCTTACAAGAAAAGAACATCCAAAACAACACCTTCATTACCTGCC

AAGAATGCTCCTTGCAAAACAAGAAACCAGTTTACTTGTGCGACAAGTGCTTCGAAGTTTACCATT
TGGAAATCAACGTCAACAGAGACAACTTCGATAAGAAGAACTTCTCCAGATTGTCCACCTTGAAG
AACTCTGTTACTACTGTTCCAATCGACAAAATGGCTCCAAAGCCATTGCAAAATGGTTACGGTTCT
AATGGTTTGAACGGTGGTTACAATACTGTTAACCCACCAATCCAACAACCATTGATGAACAATGGT
TACAACGGTTACCAACCACAACAAACACAAGTCAGAAGAAGAAGAACCGCCAATATTCAAGATG
GTTACGAAACTTCTGGTCCAGCTTTTAATTCTATGGTTCCACCACAAATGAACGGTAACATGATGG
GTCAACAAATCCCTTTGAAAAGAAGAGGTCCAGCATTGGATGATTCCGGTTTTAGATCTGATGCTC
CATCTTCTTATATTCCAGCTACTAGAGGTAGAAAAAAGTTGAACCATGGTCAAGACCAATTGCCAC
CAAGAAATCAATATGGTTACTCTGACTTGGGTCAACCTAACAACATGAATGGTTATGGTCAAGGTA
TGGGTAACAACGCTACAAACGGTTATGATAGATACTCCAGAGGTGTTGATATGAACGGTTACACT
ACATCTGATCCAGCTTACGGTAGAAATTACCAAGGTGCTGATAACTATAACAACAATCAAATCTAC
AGAGGTGTCGGTACTCAAAGAAAGTGAGCAATTCCCGGGGATCCCTCGAGCTGCGG

**Table S1. List of oligonucleotides, Related to Experimental Procedures**

| Name | 5'-3' sequence |
|---|---|
| <Primers used for genotyping of COI6 KO cells> | |
| COI6-KO-3RV2-SacII | CCGCCGCGGTTTTTAATCAGCTTAATTTAGGTC |
| COI6-KO-5FW-XhoI | GCGCTCGAGACTGTAGCTATTAATAACTATTAG |
| <Primers used for genotyping of COI7 mutants, (first round)> | |
| COI7_CasMut_cFW1 | AGTACCTTAATAATTGGATCTATCTC |
| ttCoi7_Cas9gt_RV2 | ACATATCTCTAATCTTAGCAGTCTC |
| <Primers used for genotyping of COI7 mutants, second round> | |
| COI7_CasMut_cFW1 | AGTACCTTAATAATTGGATCTATCTC |
| COI7_Mic_CasMut_cRV3 | AAAGTTAGTAATATAGCTGTTCGTGC |
| <Primers used for producing *JMJ1* KO construct> | |
| JMJ1_5f22 | CGTTTCAATGAGACCTACTTG |
| JMJ1_Nr1338 | CATAAGATAATTTGGATCAATTCTATG |
| JMJ1_Cf6613 | TGAGAGTTATTATGAATCAAACAAAC |
| JMJ1_3r7939 | GTGTCAAATTTTAGAGATTAGTGC |
| <Primers used for genotyping of JMJ1 KO cells> | |
| JMJ1_gtPCR_FW | GATTTAAATAAACAAAATTTTTTTTTGCAC |
| JMJ1_gtPCR_RV2 | AATTACCTTTTGGAATTGAAAGCTC |
| <Primers used for DNA elimination assays> | |
| bIES320-321_FW2 | GAAAAAGTATAATCTTATATATGCAAG |
| bIES320-321_RV2 | TAAAACAATTAAAGATATATACCTG |
| bIES2246-2248_FW2 | ATATTTTTAAAGTTTTAGATTGGAG |
| bIES2246-2248_RV2 | ATGCTTATTTAATTTACAAAAGGTG |
| bIES2279-2280_FW2 | TAATCATAATATTATAAATTGTGGG |
| bIES2279-2280_RV2 | GAATGTTTTTTTTTAATGAATAGAG |
| bIES381-382_FW3 | GTAAAAATAAGCAATTTAATAATTCCTAC |
| bIES381-382_RV3 | CATAAAAAGTGAAAGGTCTATACCAG |

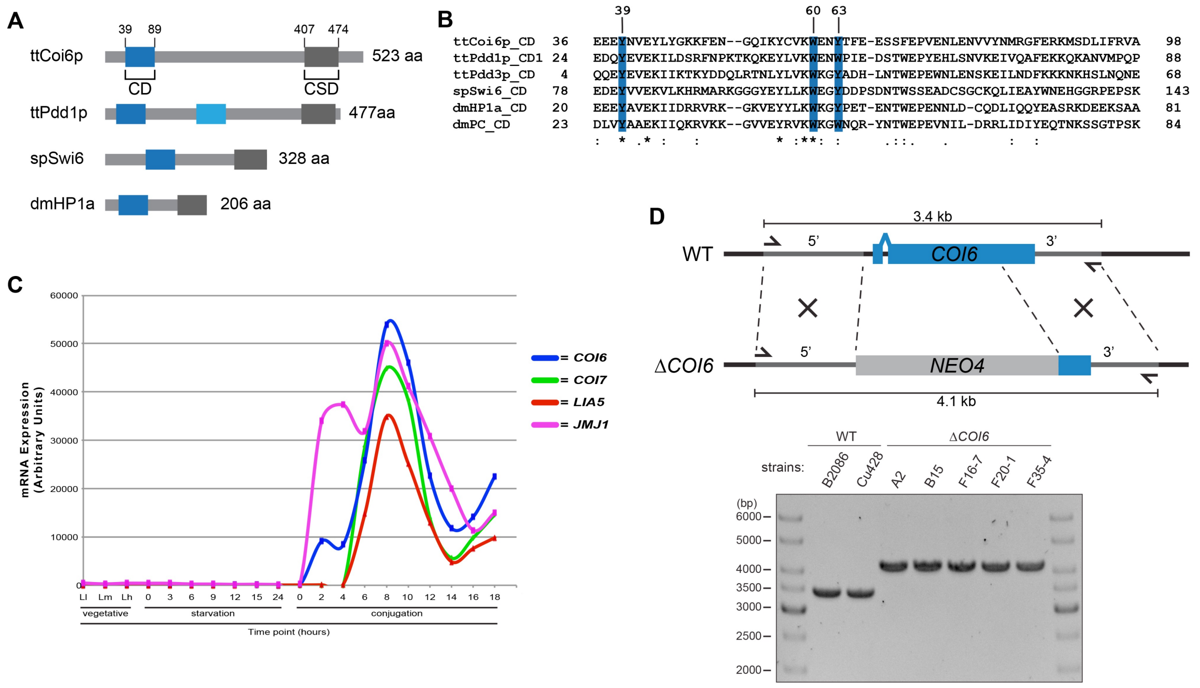


**Figure S1 (related to Figure 1). Domain architecture, expression and disruption of Coi6p**

(A) Comparison of *Tetrahymena* Coi6p and Pdd1p with other HP1 homologs (*S. pombe* Swi6 and *D. melanogaster* HP1a). The chromodomain (CD) and chromoshadow domain (CSD) are highlighted. (B) Comparison of chromodomains. The conserved amino acid residues for the aromatic cage are shaded. (C) Expression profiles of mRNAs based on publicly available microarray data (Miao et al., 2009). (D) Production of *ΔCOI6* strains. (Top) Schematic depictions of the WT *COI6* and *ΔCOI6* loci. (Bottom) Results of genomic PCR analyses of WT and *ΔCOI6* strains. The primers used are represented as arrows in the top panel.

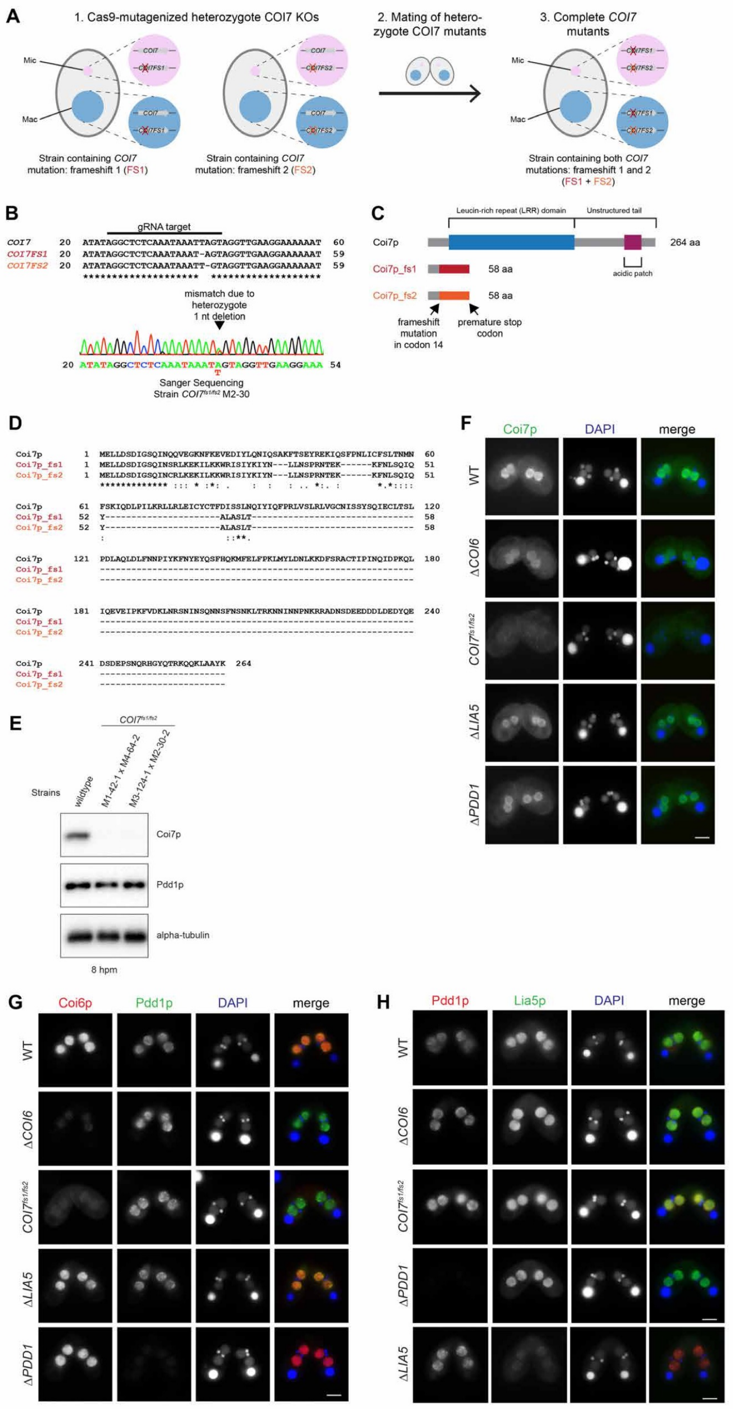

**Figure S2 (related to Figure 4). Generation of *COI7* mutant cells**

(A) Schematic representation for the genetic cross producing the transheterozygous *COI7* mutant strain $COI7^{fs1/fs2}$ from the two heterozygous *COI7* mutant strains possessing $COI7^{fs1}$ or $COI7^{fs2}$ alleles produced using CRISPR/Cas9 technology. (B) Comparison of the wild-type *COI7*, $COI7^{fs1}$ and $COI7^{fs2}$ alleles around the Cas9-targeted regions (top) and depiction of Sanger-sequencing results for the same window derived of a $COI7^{fs1/fs2}$ strain. (C, D) Schematic drawings (C) and the predicted sequences (D) of the truncated proteins produced from $COI7^{fs1}$ and $COI7^{fs2}$ alleles. (E) Western blot analysis of $COI7^{fs1/fs2}$ cells using the anti-Coi7p, an anti-Pdd1p and an anti-alpha- tubulin antibody. (F-H) Localizations of Coi7p (F), Coi6p (G) and Lia5p (H) in the indicated strains at 8 hpm were analyzed by indirect immunofluorescence staining.

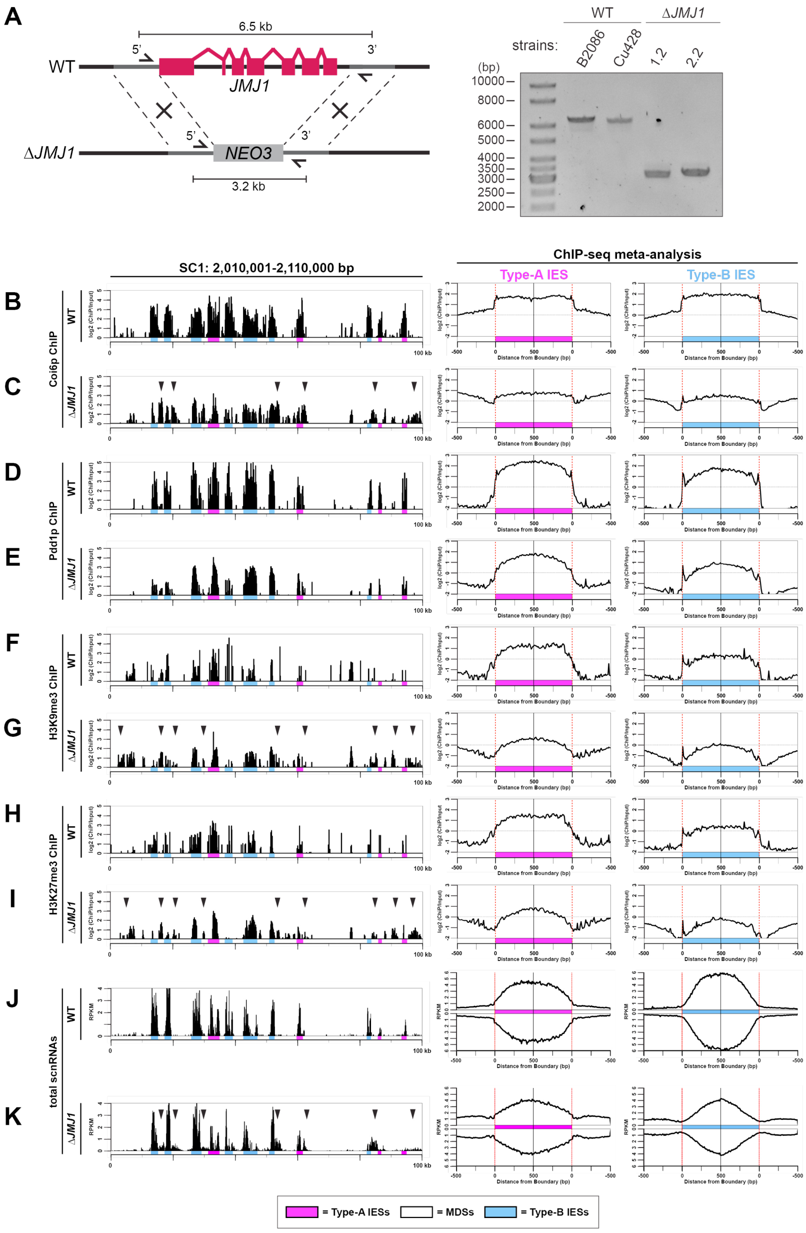


**Figure S3 (related to Figure 5). Production and analyses of *ΔJMJ1* cells**

(A) Production and verification of *ΔJMJ1* strains. (Left) Schematic depictions of the WT and *ΔJMJ1* allele of *JMJ1* loci. (Right) Results of PCR analysis of genomic DNA from WT and *ΔJMJ1* strains. The primers used for the PCR are represented as arrows in the left panel. (B-I) The chromosomal localizations of Coi6p (B, C), Pdd1p (D, E), H3K9me3 (F, G), and H3K27me3 (H, I) in WT (B, D, F, H) and Δ*JMJ1* (C, E, G, I) cells at 12 hpm were analyzed by ChIP-seq. Sequence reads were mapped to a 100-kb genomic region with 100-bp bins (left) or to compiled 500-bp sequences inside and outside of the boundaries of Type-A and Type-B IESs with 10-bp bins (right), and the mapped and normalized read numbers from ChIP-seq were divided by the corresponding numbers from Input. Type-A and Type-B IESs were marked in magenta and blue, respectively. (J-K) Small RNAs from WT (B, D, F, H, J) and Δ*JMJ1* (C, E, G, I, K) cells at 12 hpm were sequenced, and 26-32-nt RNAs (scnRNAs) were mapped to a 100-kb genomic region with 100-bp bins (left) or to compiled 500-bp sequences inside and outside of the boundaries of Type-A and Type-B IESs with 10-bp bins (right). In the meta-analyses, numbers of sense and anti-sense strand mapped scnRNAs are shown on the top and bottom of each graph, respectively. Arrowheads indicate MDS regions in which the ectopic production of scnRNAs was detected.

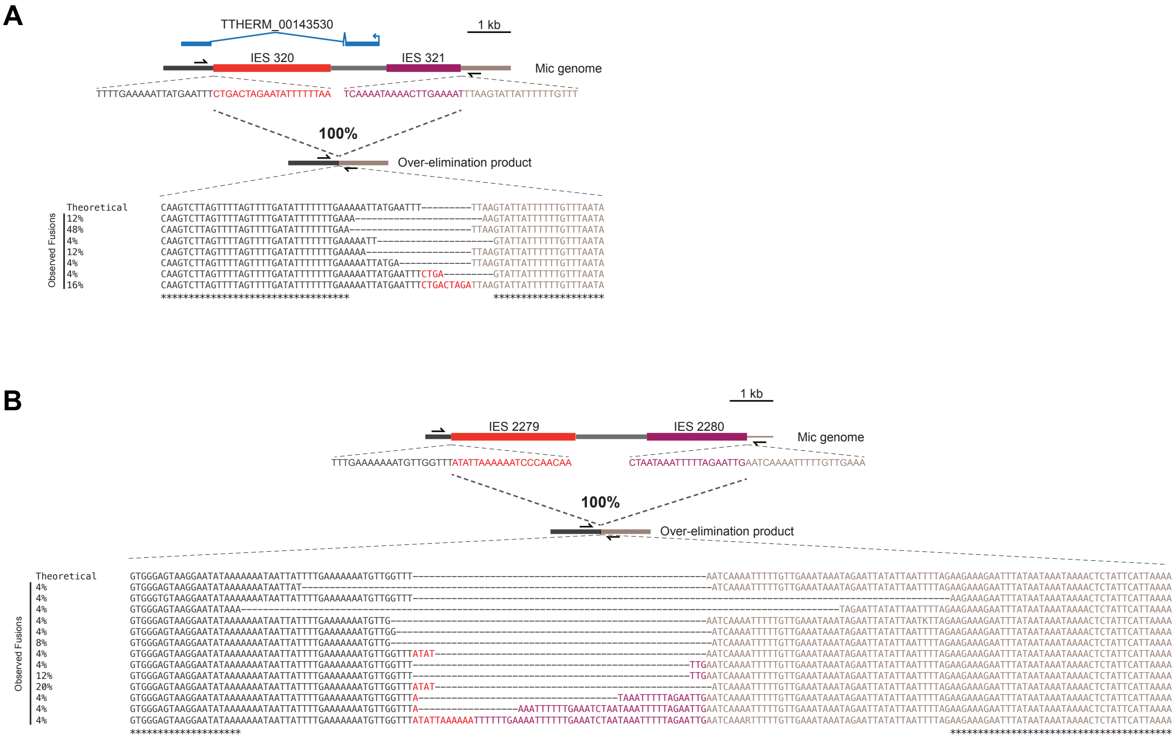


**Figure S4 (related to Figure 6). Sequence analyses of abnormal elimination products in *ΔCOI6* cells**

The shorter PCR products in *ΔCOI6* cells at 24 hpm from IES 320-321 (A) and IES 2279-2280 (B) loci (shown in Fig. 6B and C) were cloned, and at least 20 clones from each product were sequenced. Predicted ectopic elimination products (top) and the sequences of PCR products were aligned.

**References for Supplemental Information**

Loidl, J., and Scherthan, H. (2004). Organization and pairing of meiotic chromosomes in the ciliate *Tetrahymena thermophila*. J Cell Sci *117*, 5791–5801.

Miao, W., Xiong, J., Bowen, J., Wang, W., Liu, Y., Braguinets, O., Grigull, J., Pearlman, R.E., Orias, E., and Gorovsky, M.A. (2009). Microarray analyses of gene expression during the *Tetrahymena thermophila* life cycle. PLoS One *4*, e4429.

Miller, J., and Stagljar, I. (2004). Using the yeast two-hybrid system to identify interacting proteins. Methods Mol. Biol. *261*, 247–262.

Song, X., Gjoneska, E., Ren, Q., Taverna, S.D., Allis, C.D., and Gorovsky, M.A. (2007). Phosphorylation of the SQ H2A.X motif is required for proper meiosis and mitosis in *Tetrahymena thermophila*. Mol Cell Biol *27*, 2648–2660.
